# Biopurification using non-growing microorganisms to improve plant protein ingredients

**DOI:** 10.1038/s41538-024-00290-x

**Published:** 2024-07-31

**Authors:** Avis Dwi Wahyu Nugroho, Saskia van Schalkwijk, Sabri Cebeci, Simon Jacobs, Wilma Wesselink, Guido Staring, Soenita Goerdayal, Andrei Prodan, Ann Stijnman, Emma Teuling, Kerensa Broersen, Herwig Bachmann

**Affiliations:** 1 Systems Biology Lab, A-LIFE, AIMMS, Vrije Universiteit Amsterdam, Amsterdam, The Netherlands; 2 Microbiology department, NIZO food research B.V, Ede, The Netherlands; 3 Food department, NIZO food research B.V, Ede, The Netherlands; 4 Applied Stem Cell Technologies, University of Twente, Technical Medical Centre, Enschede, The Netherlands; 5 Present Address: CJ Research Centre Europe, Wageningen, The Netherlands; 6 Present Address: Single Cell Discoveries, Utrecht, The Netherlands

**Keywords:** Industrial microbiology, Agriculture

## Abstract

Securing a sustainable global food supply for a growing population requires a shift toward a more plant-based diet. The application of plant-based proteins is therefore increasing, but unpleasant off-flavors complicate their use. Here, we screened 97 microorganisms for their potential to remove off-flavors in a process with limiting amounts of fermentable sugar. This allowed the production of a more neutral-tasting, purified food ingredient while limiting microbial growth and the production of typical fermentation end products. We demonstrate that various lactic acid bacteria (LAB) and yeasts remove “green” aldehydes and ketones. This conversion can be carried out in less than one hour in almond, pea, potato, and oat proteins. Heterofermentative LAB was best at aldehyde and ketone neutralization with minimum de novo formation of microbial volatiles such as ethylacetate (sweet, fruity) or alpha-diketones (butter- and cheese-like). While sensory properties were improved, changes in protein solubility, emulsification, foaming, and in vitro digestibility were limited.

## Introduction

Plant-based food is currently the largest source of non-animal-derived, alternative protein with a continuous growth in global consumption^1^. Regarding price parity, technological maturity, regulatory support, and consumer acceptance^2–4^, plant proteins score highest in comparison with other alternative proteins such as microbial-, recombinant- or tissue culture-derived proteins. Overall, a plant-based diet is often described to be more sustainable and healthier than a meat-based diet^5–9^. To successfully replace animal products, sensory characteristics, and overall liking are crucial, and most consumers are unwilling to compromise on taste^10,11^. However, the use of plant-protein is regularly hampered by off-flavors that are described as “green”, “grassy”, and “beany” which are the result of the presence of common volatiles^12,13^. In legumes, off-flavor-associated volatiles are contributed by saturated aldehydes (e.g., hexanal), unsaturated aldehydes (e.g., cis-3-hexenal), and methoxypyrazines^14^. The formation of such “green” aldehydes is a result of fatty acid oxidation, particularly polyunsaturated fatty acids (PUFAs). Plant seeds accumulate these compounds as nutrient reserves^14^, and they occur in their cell membranes. Hence, these off-flavor precursors can be found in many plant-based raw materials, including non-oleaginous materials, such as potato tubers^15^. In plant-based foods, lipoxygenase (LOX)-catalyzed degradation of PUFAs is believed to be the main formation pathway of off-flavors^16^. Strategies to prevent off-flavor formation include LOX-null plants^15,17,18^, heat-induced LOX inactivation, enzymatic removal of precursors (phospholipids)^19^, or solvent treatments. However, such strategies often adversely affect protein techno-functionality, e.g., solubility^20^. The microbial reduction of off-flavors is an alternative approach that is mild and robust and typically applied through conventional fermentation^21–24^. Depending on the processing conditions, plant-based products can be at risk of outgrowth of resident spoilage and pathogenic microbes^25,26^. Fermentation can prevent such outgrowth, but the production of organic acids, ethanol, microbial volatiles, or the degradation of plant proteins results in an altered sensory profile. A common bias exists that microbial fermentation obliterates protein functionality, while improvements have been widely seen when a methodical approach is taken^27^.

Here, we investigated an alternative approach where we design and test a process in which the production of fermentation end-products is limited, but the desired metabolic conversions of off-flavors are still active. We consider this a biopurification process, which we carried out with diverse lactic acid bacteria (LAB) and yeasts to lower the amount of off-flavor molecules in almond, oat, pea, and potato proteins. The identified strains allowed for a rapid (≤1 h) reduction of aldehydes and ketones with negligible production of microbial (off) flavors or organic acids and minimum alterations of protein techno-functionality.

## Results

### Biopurification allows volatile degradation in the absence of a carbon source

To study the role of microbial conversion of molecules occurring in the seeds of four diverse plant families, we characterized volatile compounds present in almond (stone fruit), oat (gras), pea (legume), and potato (tuber) protein and found considerable overlap between substrates (Supplementary Information S1). Detected differences are partially explained by the source of the raw material or are the result of the protein purification process itself (Supplementary Information S2). Analysis of the sugar composition (Supplementary Information S3) shows that no sugars were detected in the pea and potato protein isolates used in this study. The oat protein concentrate used contained a small amount of sucrose in the powder (6 mg/g), while the almond protein concentrate contained a higher amount of sucrose (94 mg/g) as well as small amounts of stachyose (4 mg/g), raffinose (10 mg/g), and mannose (6 mg/g). In addition to the measured sugars, carbon may be present in the form of a wide range of glycosides naturally present in plant protein ingredients, e.g., phenolic compounds and saponins^28,29^, and if liberated through enzymatic processes, these sugar groups may facilitate the growth of microorganisms. Microbial sugar catabolism may be crucial for volatile reduction since it regenerates the required redox cofactors in the cell (e.g., NAD(H))^30^. Under growing conditions, an increase in the number of cells may enhance volatile reduction. However, high acidity or ethanol production may eventually halt cellular activity^31^, and enzyme activities might alter protein functionality. This raises the question of whether volatile reduction can occur in a situation where growth is limited due to e.g., a lack of fermentable carbon^32,33^.

**Fig. 1:**
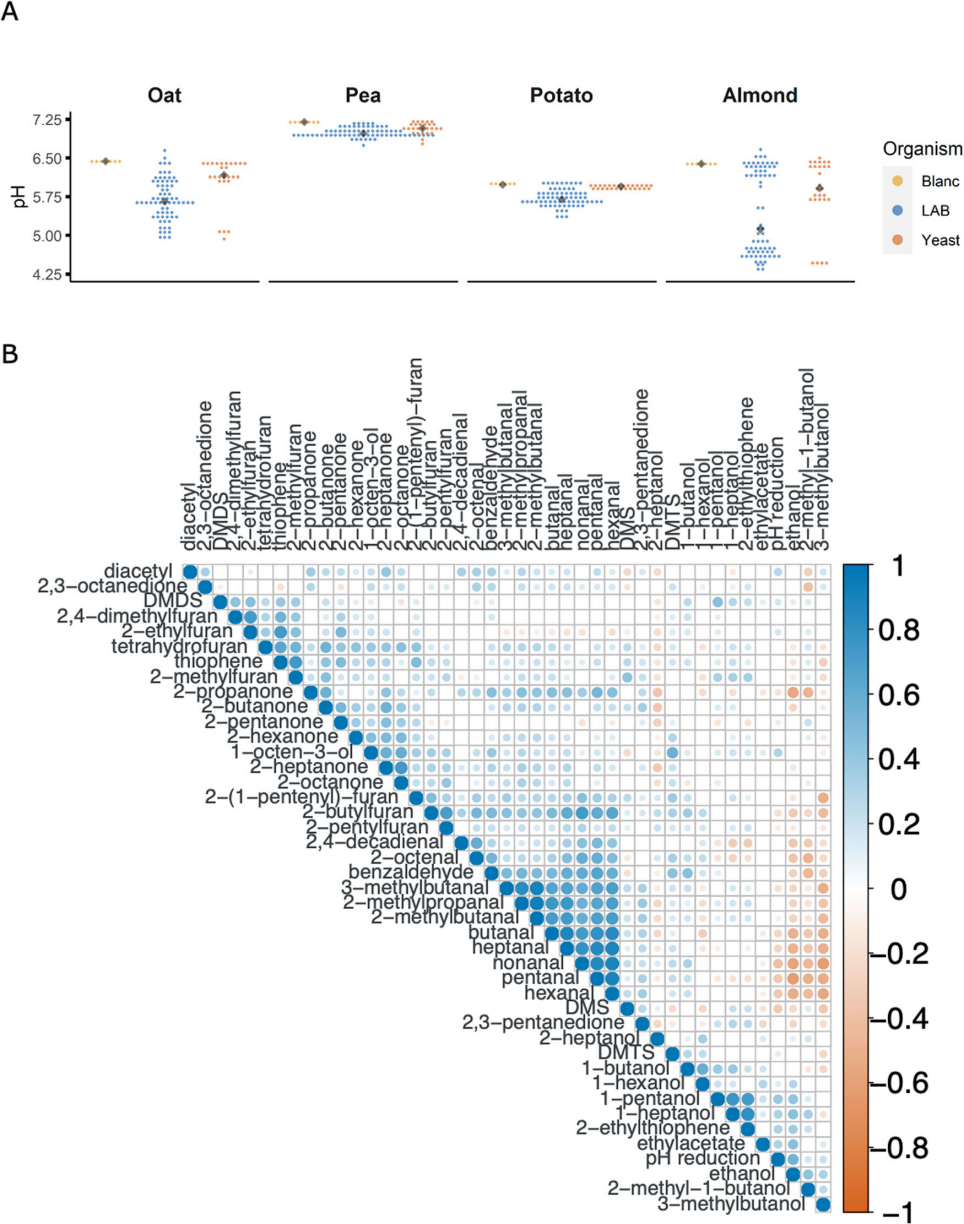
Four plant protein solutions (no sugar added) were incubated with 70 LAB and 27 yeast strains for 24 h. The final pH values are shown in panel (**A**). Each dot corresponds to a sample with an individual organism (differentiated by colors). Black diamond-shaped dots correspond to the median of the dataset. Correlations of measured volatiles and pH reduction (using non-fermented samples as reference) at the end of sample incubation are shown in panel (**B**). Correlation values were calculated as Spearman’s rho based on the values obtained from biopurification of hour substrates and visualized in varying circle sizes and a color gradient. Blank cells represent non-significant correlations (*p*-value < 0.05).

**Fig. 2:**
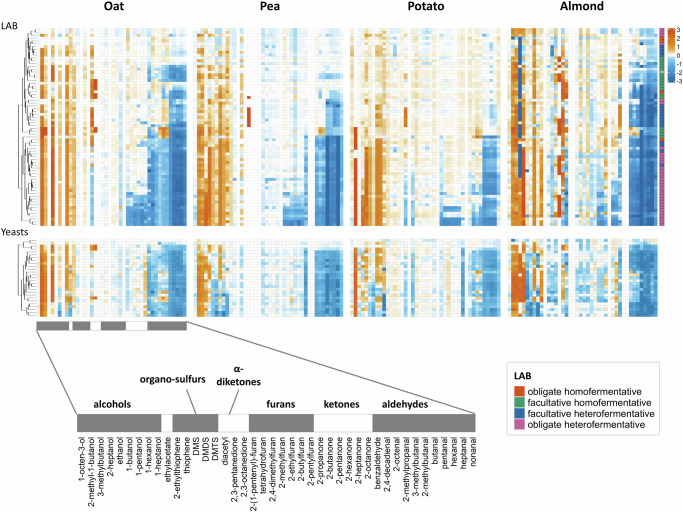
Heatmap of volatiles in different plant protein ingredients following biopurification with LAB (upper panels) or yeast (lower panels) as measured by HS-SPME-GC-MS after 24 h of incubation. Each row on the *y*-axis represents the biopurification profile from one strain. Volatiles (columns) were manually grouped per compound class as indicated in gray and white blocks at the bottom. Heatmap colors are log10-transformed fold changes compared to the corresponding non-inoculated control samples. The row color bar of the upper LAB panel represents fermentation profiles based on lactic vs mixed acid productions which are taken or deduced from literatures^47,48^. Extended data are available in Supplementary Information S6 and S12.

To investigate this, we incubated a diverse set of microorganisms, mainly of food origin, with plant protein solutions in either the presence or the absence of added sugars (Supplementary Section 4). Following 24 h of incubation, the pH values of the protein solutions were determined as a proxy of sugar utilization (acidification) and/or microbial growth. We would like to note that the assumption that a pH decrease coincides with growth might not always be fully accurate for some of the yeast strains used. Without the addition of sugar, limited pH changes across samples were seen in pea and potato protein isolates (Fig. 1A). In almond and oats, the pH decreased from 6.4 to a minimum of 4.3 and 5.0, respectively, independent of whether yeast or LAB was used. The pH drop is in line with the measured levels of sugars, but it is also obvious that not all strains led to the same pH decrease. The data further show that a declining pH coincides with lower levels of most ketones and aldehydes. At the same time, the alcohols increased (Fig. 1B), and sugar utilization correlated with ethyl-acetate production (Supplementary Information S4). Together, these results demonstrate the potential of lowering plant-derived volatiles using microbial metabolism. However, while sugar utilization by microorganisms improves the number of aldehydes and ketones that can be reduced, a more detailed analysis shows that individual strains are able to reduce these compounds, in e.g., oat and pea samples where limited acidification and, therefore, limited growth occurred. For instance, in peas, hardly any acidification occurred with yeasts or LAB (Fig. 1), but many of these organisms were able to reduce aldehydes and ketones (Fig. 2). This suggests that the potential for biopurification is strain-specific.

### Heterofermentative LAB reduced aldehydes and many reduced ketones as well

A limited set of microorganisms have been reported before for their ability to neutralize off-flavors in multiple plant proteins^21,23,24,34^. There are indications that the extent of the neutralization of common off-flavors during the fermentation of plant protein ingredients is strain-dependent and that the spectrum of activities overlaps within specific phylogenetic clades of microorganisms^24^. However, there is no data available on the extent to which such conversions are possible when the carbon sources in the substrate are limited. Our dataset with 70 lactic acid bacteria and 27 yeasts combined with four substrates allowed us to address this question by comparing the relative abundance of volatiles at the end of the incubation (Fig. 2). The observed overall spectrum of volatile reduction can be categorized based on volatile classes, particularly aldehydes, and ketones. Despite differences in protein matrices, volatile reductions were strikingly consistent, particularly for yeasts and remarkably for obligate heterofermentative LAB. Both groups of microorganisms are known for their high constitutive expression of alcohol dehydrogenases (ADH) and the production of ethanol as one of the end metabolites of pyruvate dissipation. Aldehydes and ketones were effectively reduced by obligate heterofermentative strains, while some strain- and species-dependent variations were found for other LAB^35,36^. In all cases, the reduction of aldehydes or ketones was accompanied by the concomitant formation of the corresponding alcohols. Notably, three LAB species (*Lb. buchneri, Lb. parabuchneri*, and *Lb. brevis*) effectively reduced unwanted plant-derived volatiles with minimum formation of microbial volatiles (Supplementary Information S5) and limited pH reduction. Such microorganisms fulfill the desired requirements of protein biopurification i.e., the production of a neutral-tasting plant-protein ingredient. The obligate heterofermenters seem to be the most promising group of microbes with a spectrum of reduction of both aldehydes and ketones (Fig. 2 and Supplementary Information S6). The LAB and yeast strains that show the highest potential for biopurification can be related to elevated ADH expression levels.

### Degradation of aldehydes is rapid and conferred by ADH

**Fig. 3:**
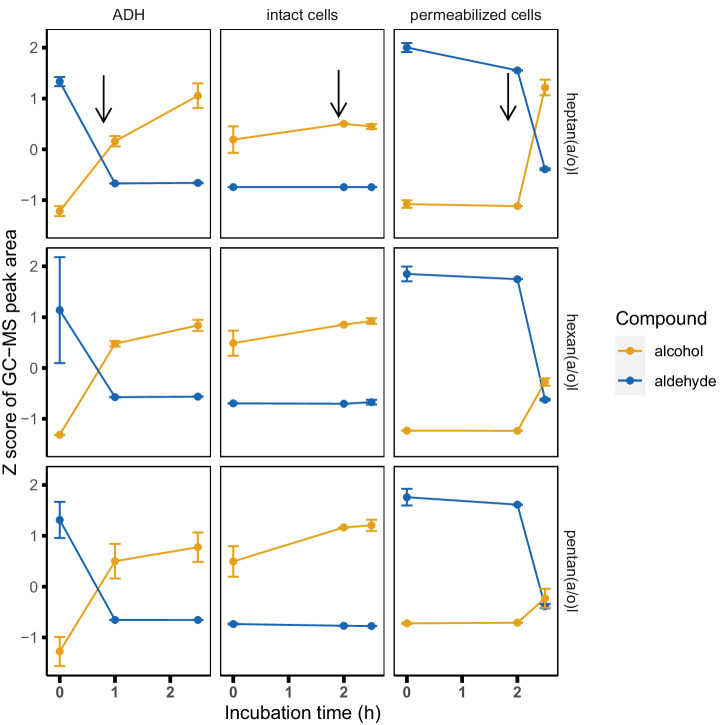
Rapid reduction of aldehydes (blue) to their corresponding alcohols (orange). Isolated ADH, intact LAB cells, and permeabilized LAB cells (columns from left to right) were used as biopurifying agent. The cell concentration used was at ~1E + 08 cells/ml. Error bars represent the standard deviation of (biological) replicates (*n* = 3 for ADH and *n* = 2 for cells). NADH was added after 1 or 2 h of incubation, as indicated by black arrows in the upper row. Note: when using intact cells the aldehyde was reduced in the time between preparing and freezing the samples. See Supplementary Fig. 9 for an extended version.

Our initial screening (Fig. 2) involved the incubation of protein solutions for 24 hours as an end-point measurement. To test how fast biopurification can be achieved, we studied the use of high inoculation densities (~1E8 cells/mL) with a well-performing, biopurifying strain (LAB11—*Leuconostoc mesenteroides)*. In addition, we investigated whether ADH activity alone was sufficient for aldehyde reduction. We did this by either using a commercial mixture of ADHs isolated from *Saccharomyces cerevisiae* or alternatively by working with permeabilized cells of LAB11. All three conditions were used to test the degradation of aliphatic aldehydes in pea protein. As the conversion from aldehyde to alcohol by isolated ADH and possibly permeabilized cells relies on exogenous NADH, we added NADH 1 or 2 h after the incubation was started. Consistently the results show that the levels of aldehydes at the start of the incubation in permeabilized cells and ADH-treated samples were high, and their reduction was observed only after NADH addition (Fig. 3). This shows the NADH dependency of the conversion reaction with purified ADH, confirming that ADH is the conferring agent of the reaction. Low levels of aldehydes could be observed almost immediately after NADH addition. Additional incubation or supplementation with NADH did not lead to further aldehyde reduction (Supplementary Information S7), indicating rapid completion of the reaction. Interestingly, in samples where intact cells were used, the level of aldehydes already reached their lowest values at the first measurement point, and longer incubation or the addition of NADH did not lead to further aldehyde reduction. In this case the levels of aldehydes reached similarly low values as what we found with purified ADH or permeabilized cells after NADH addition. This indicates that intracellular NADH equivalents in strain LAB11 are sufficiently supplied in cells incubated in a pea-protein isolate without a carbon source. Under all conditions, the reduction of the aldehyde resulted in a high level of the corresponding alcohol derivatives which is in line with the described bioconversion by ADH. Altogether, the neutralization of aliphatic aldehydes can be very rapid when incubating the substrate with 1E8 cells/ml. In addition to the removal of aldehydes and ketones some strains lead to lower furan levels, especially in pea and almond. Some yeasts also lead to a decrease in sulfur compounds, which was particularly pronounced in almonds as a substrate (Fig. 2).

### Optimization of biopurification through high dry matter content fermentations and preculture conditioning

For the development of sustainable biopurification processes, water addition should be minimized to decrease the energy needed to, e.g., produce a protein powder ingredient following biopurification. In dedicated experiments, we investigated combinatorial options where we varied inoculation densities and incubation times in a pea protein matrix with a high dry matter content. We used a dry matter content of 40%, which gave a dough-like texture in which we expected to have sufficient water available for microbial activity. The results showed that also at 40% dry matter content, the aldehyde reduction was almost complete in less than 30 min after inoculation (first measurement) with 1E8 cell/ml or more, while the process took up to 24 h when the inoculation density was decreased to 1E6 cells/ml (Supplementary Information S8). The absence of a detectable decrease in pH or microbial growth during the first hour points towards a microbial lag phase (Supplementary Fig. 12) during which biopurification occurs.

**Fig. 4:**
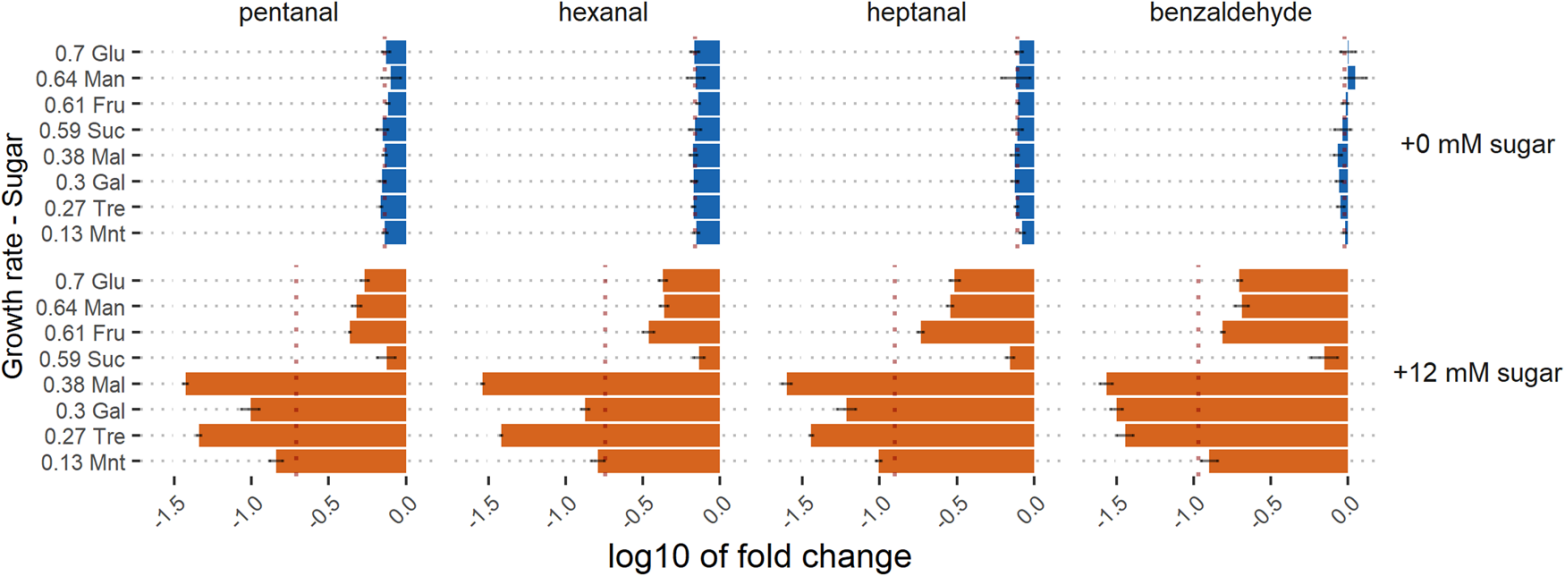
Fold-change (Log10) of volatiles pea samples biopurified with *L. cremoris* MG1363 in comparison to sterile unfermented samples. *Y*-axis shows growth rate (/h) and 3-letter initial of sugar added during preculture: glucose (Glu), mannose (Man), fructose (Fru), sucrose (Suc), maltose (Mal), galactose (Gal), trehalose (Tre), and mannitol (Mnt). Blue and orange bars show volatile changes after incubation without and with sugar added to the pea protein suspension respectively. Error bars show the standard deviation of the mean (*n* = 3). See Supplementary Fig. 4 for more data.

While heterofermentative strains were shown to be efficient for biopurification, these organisms are often not the first choice in industrial applications. For this reason, we explored a strategy to increase ADH levels in the typically homofermentative and easy-to-culture organism *L. cremoris*. *L. cremoris* is known to undergo a metabolic shift from homolactic to mixed acid fermentation when cultured at lower growth rates^37,38^. This comes with higher expression levels of the ADH enzyme. We tested whether varying the preculture growth rate through incubation on different carbon sources influences aldehyde reduction when the cells are subsequently used for biopurification. The results show that supplying a sugar that leads to a slow growth rate allows a homofermentative lactococcus to significantly increase the removal of aliphatic aldehydes (Fig. 4, Supplementary Information S4). This opens opportunities for the optimization of homofermentative organisms to efficiently be applied for biopurification.

### Sensory analysis confirms biopurification-induced volatile changes

To assess the remaining odor-active compounds after biopurification of pea protein, they were identified using GC-olfactometry. While decreased in peak area (GC–MS), odor-active compounds were still perceived and included sulfury (sulfur dioxide, methanethiol, methional, dimethyltrisulfide), fresh, green, grassy (hexanal, hexanol), creamy buttery, cheesy (diacetyl, 2,3-pentanedione, 2-heptanone), cocoa, malty (3-methylbutanal), fatty, green, cucumber (octanal, nonanal, 2-heptenal, 2-octenal), mushroom (octen-3-ol, 1-octanol, 3,5-octadien-2-one), sweet vanilla (vanillin), sweet roast (furfural), pea, green (methoxypyrazines), fatty, and coconut (gamma-undecalactone) (Supplementary Table S5).

**Fig. 5:**
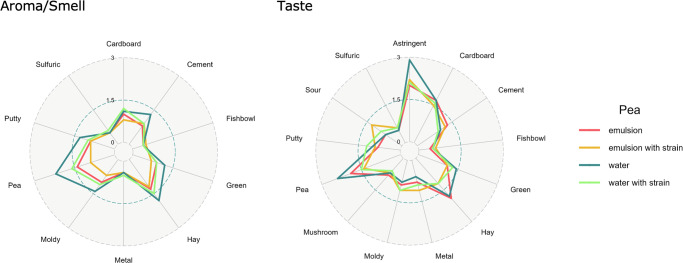
Spider web representation of aroma and taste attributes of the pea protein beverage (water-based) and the milk analog (emulsion-based) before (red and blue) and after fermentation (orange and green). Results are the average of scores obtained by ten panelists.

To investigate whether biopurification leads to perceivable sensory changes in relevant applications, a CATA (Check All That Applies) analysis was carried out in 2 beverages. As the food matrix influences flavor and release, the sensory analysis was performed in a water-based (beverage) matrix and in an emulsion-based (milk analog) matrix. An ANOVA analysis showed that biopurification significantly reduced the pea attribute (*p* = 0.04, d*f* = 3) albeit differently in the two matrices (Supplementary Information S9). Other attributes, including sour, did not change significantly (Fig. 5). The changes in sensory perception can be related to biopurification-induced compound changes identified with GC–MS and GC–O, such as hexanal and 2-octenal (Supplementary Information S9). Further optimizations through choosing combinations of strains that can reduce the remaining aroma-active compounds as observed with GC–O could be a next step.

### Biopurification has limited effects on in vitro digestibility and techno-functionality

Industrial applications of proteins require specific functionality, and the process of biopurification may affect these properties. For example, microbial membrane-bound or secreted proteases may affect protein digestibility, alter techno-functionality, or produce bitter peptides^27,39,40^. To investigate whether the digestibility of plant proteins was affected by biopurification, we subjected pea protein that had been biopurified with three selected strains to an INFOGEST in vitro digestion paradigm, mimicking the conditions of gastrointestinal processing^41^. Peptide analysis showed that susceptibility to gastrointestinal processing was largely not affected upon biopurification. The release of amino acids in the same samples showed a clear strain dependency allowing for a targeted process design (Supplementary Information S10). For *L. mesenteroides* but more so for the yeast *Pichia manshurica* the effects on amino acid concentrations after biopurification and in vitro digestion were limited. Overall, the susceptibility of pea protein to gastrointestinal digestion is largely unaffected upon exposure to the strains chosen for biopurification.

**Fig. 6:**
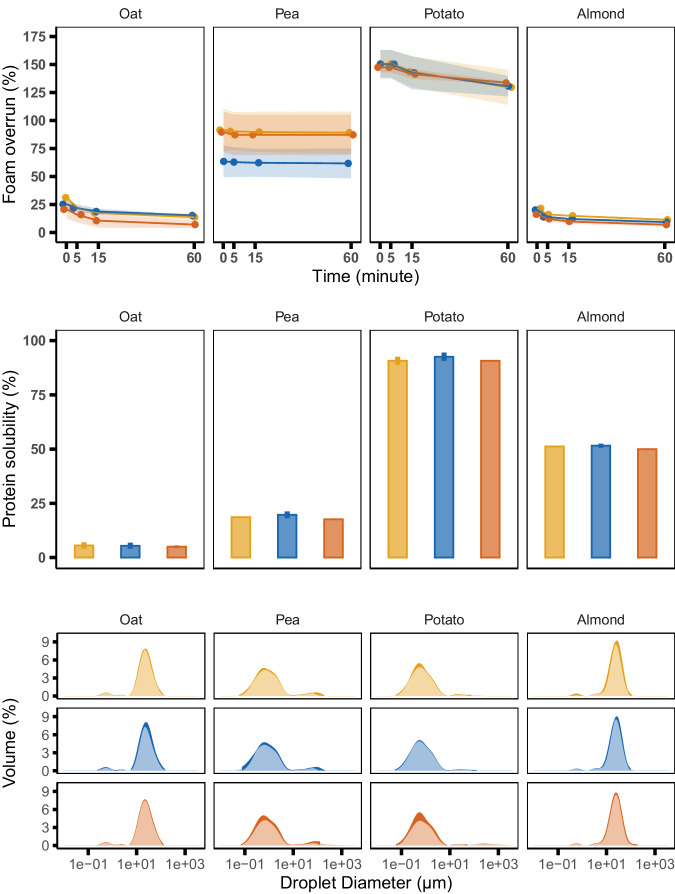
Techno-functionality of non-sterilized plant-protein ingredients following biopurification using the lactic acid bacterium *L. buchneri* (LAB98) (light orange) the yeast *Pichia deserticola* (Yeast33) (red) or the non-inoculated control (blue). Freeze-dried, biopurified protein samples (*n* = 2) were resuspended and analyzed for foaming (top panel), solubility (middle panel), and emulsion droplet size (bottom panel). Error bars/shaded areas indicate standard deviations of the mean.

Studies on plant protein fermentation reported that incubation times exceeding 10 h negatively affected functional properties associated with the degradation of proteins or peptides^21,23^. To test for changes in protein techno-functionality, foaming, emulsification, and protein solubility were compared for pea protein solutions before and after biopurification (Fig. 6 and Supplementary Information S11). Overall, foam overrun, total soluble nitrogen, and emulsion droplet size distribution were not significantly affected by biopurification. One exception was the pea protein isolate, where biopurification by LAB or yeast resulted in a 25% increase in foaming ability and stability. Aside from biopurification the results showed significant differences between the techno-functionality of various protein sources. Overall, foaming properties may be slightly improved and other properties remain unaltered by off-flavor biopurification.

## Discussion

Our initial hypothesis, that carbon-limited/non-growing microbial cells can carry out desired conversions in plant-based fermentations, was based on the fact that non-growing LAB can have significant metabolic activity^42^. This concept also applies to the use of dairy adjunct cultures. While we could show the importance of ADH under non-growing conditions, it is relevant to mention that ADH can occur as a bifunctional enzyme, also leading to the conversion of aldehydes to carboxylic acids and eventually to esters^35,36,43^. In our case, we did not observe other aldehyde-derived products, but even if that were the case, such products should be less perceivable based on their lower volatility. The removal of off-flavors does not necessarily guarantee the absence of subsequent off-flavor formation during further processing and storage of a product since the precursors of unwanted molecules may still be retained. This indicates that the position of biopurification during processing will be of importance. For aliphatic aldehydes, precursor removal has been demonstrated using (phospho-)lipases^19,43^. Such enzymes are described in lactobacilli^35,36,44,45^, and their application remains to be studied for biopurification. GC-olfactometry and the CATA analysis indicated that after the removal of the “green” aldehydes, other aroma-active compounds can still contribute to sensory off-flavor attributes. For instance, the high detection threshold for pyrazines with GC–MS leads to us not reporting any here, but the sensory data suggests that pyrazines might be unmasked following biopurification. Pyrazine-converting microorganisms have been described^46^. Their inclusion might allow further biopurification.

The option of conditioning facultative homofermentative lactic acid bacteria to increase desired enzyme activity opens opportunities for strains already in use in the industry. While the presence of ADH is a prerequisite for aldehyde reduction, we cannot exclude that physiological differences, such as intracellular NAD^+^/NADH ratios between homo- and heterofermentative organisms, are determining the conversion rates. Fast conversion rates allow to minimize the risk of outgrowth of pathogenic and spoilage organisms. These are often spore-forming organisms e.g., bacillus species present in raw materials^25^. For their inactivation, typically, high-temperature treatment is used, but this is detrimental to the native plant-protein conformation and its techno-functionality. A reduced need for hurdles to prevent the outgrowth of spoilage organisms will, therefore, benefit techno-functionality and in vitro digestion. The rate of off-flavor removal depends on the inoculation density. An estimate for the biopurification of one metric ton of protein concentrate treated in a process with 40% dry matter (weight/volume) (2500 l), using 10^7^ bacterial cells/ml (see also Supplementary Information 8), indicates that 10 l of fermentation broth with a cell density of 2.5 × 10^9^ cells/ml are required. For industrial implementation, optimizations to minimize the number of cells, inoculation time, and position in the overall process will still be needed. For the inactivation of the organisms after biopurification a pasteurization step is typically sufficient because the used organisms are heat sensitive.

While our results show that strains with specific desirable properties can be identified, screening processes will certainly benefit from a more rational approach. By using genome-based enzyme and pathway prediction analyses, the number of screened organisms for a particular biopurification purpose might be narrowed down. However, this approach is currently still restricted by the limited knowledge of pathways involved in the microbial degradation of undesired plant-derived molecules. Harnessing microbial biodiversity for the improvement of plant proteins in combination with the right processing conditions is likely to play an important role in making future plant-based foods more palatable and thereby increasing consumer acceptance. This does not only count for plant-based food but also for current developments in foods based on recombinant protein (precision fermentation) or cultured meat, where for economic reasons, the addition of plant proteins is becoming increasingly important.

## Methods

### Strain and cultivation conditions

A total of 70 lactic acid bacteria and 27 yeast strains from the NIZO culture collection were used. M17 or MRS media were used for routine cultivations of lactococci and lactobacilli, respectively. YPD medium was used for the cultivation of yeasts. Details of the strains and conditions are available in Supplementary Information S12. Unless specified otherwise, all media were supplemented with 0.5% glucose. LAB cultivations prior to biopurification experiments were performed either in 10 ml tubes or 96-well microplates without shaking, while yeast cultivations were performed with 5 ml medium in 50 ml tubes with shaking at 250 rpm. Lactobacilli, streptococci, and lactococci were incubated for 24 h, while leuconostoc and yeast were incubated for 48 h before use. Once fully grown, the cells were harvested and washed with 1 volume of PBS (phosphate-buffered saline) at pH 7.

### Plant protein preparation

Plant protein ingredients used were pea protein isolate (Pisane C9, Cosucra), potato protein isolate (Solanic 100, Avebe), almond protein concentrate (Blue Diamond), and oat protein concentrate (PrOatein, Lantmännen). Protein powders were resuspended in RO water at 10% w/w for all proteins, except for potato protein, which was resuspended at 5% w/w. Autoclave sterilization of resuspended protein solutions was performed at 110 °C for 15 min to ensure inactivation of contaminants present in the raw material. For the experiment with high dry matter content a dough-like consistency with 40% dry matter of pea protein was generated. Pea protein was heat treated for 5 h at 140 °C (dry heat) prior to the preparation of the 40% dry matter substrate.

### Biopurification with cells

For biopurification sterilized protein solutions were supplemented with washed cells. During screening in 96-well microplates, a 100-fold dilution of fully-grown inoculum was used. This equates to approximately 1E + 07 cells/ml for bacteria and 1E + 06 cells/ml for yeasts. Initial screening was done as single measurements. Sample preparation for sensory and techno-functionality assays was performed with cell densities standardized to approximately 1E + 08 cells/mL for LAB and 1E + 07 cells/ml for yeast. Biopurification was performed at 30 °C for 1 h, unless otherwise specified.

### Biopurification with permeabilized cells and enzyme preparations

For biopurification with permeabilized cells, fully-grown *L. mesenteroides* (LAB 11) were incubated in 50% ethanol for 15 min, and subsequently washed with 1 volume of PBS. Plant protein was incubated with a final concentration of 1E + 08 cells/mL. For biopurification with ADH isolated from *S. cerevisiae* (300 U/mg, Sigma Aldrich), the enzyme was resuspended in PBS (pH 7) and added at a final concentration of 22.5 U/mL. NADH (Sigma Aldrich) was resuspended in demineralized water and added at the indicated time to a final concentration of 0.4 mM in plant proteins.

### GC–MS analysis

Aliquots for GC–MS analysis were stored at −20 °C until analysis. Volatile analysis was performed using headspace solid phase microextraction (HS-SPME) carried out in combination with gas chromatography/mass spectrometry (Fisons, USA) as previously described^31^. Samples of starting points at time zero (T0) took practically 10–30 min between mixing the protein powders with strains and storing them in the freezer. This is relevant as during this sampling period many compounds were degraded when high cell densities were used.

### Sensory analysis

Samples for sensory analysis were prepared as an aqueous drink and a milk analog (an oil-in-water emulsion). For details, see supplementary information.

### Data analysis

For data analysis and visualization, Rstudio (2022.07.1 + 554 with R version 4.1.2) and the packages tidyverse v1.3.2, ggplot2 v3.3.6, ComplexHeatmap v2.10.0 and corrplot v0.92 were used.

### Supplementary information

Supplementary Information

## Data Availability

The work presented in this manuscript was carried out in a TKI-supported public-private partnership. The majority of the microbial strains used are owned by NIZO Food Research, a privately held contract research organization. Access to strains, code and data used can be obtained by contacting the corresponding author or info@nizo.com. For commercial use license agreements will need to be negotiated. For academic use NIZO will need to be acknowledged in all communication and free access to generated results needs to be granted. For microbial strains conditions/MTAs will apply.

## Code availability

Access to the code used for data analysis can be obtained by contacting the corresponding author or info@nizo.com.
