# Supplementary material for: Biopurification using non-growing microorganisms to improve plant protein ingredients
Source: NPJ Sci Food. 2024 Jul 31;8:48. doi: 10.1038/s41538-024-00290-x (PMC11291906; doi:10.1038/s41538-024-00290-x)
Supplement: Supplementary file 1 — Supplementary Information [file 41538_2024_290_MOESM1_ESM.pdf]

Supplementary Information

Biopurification using non-growing microorganisms to improve plant protein ingredients.

Avis Nugroho[1#], Saskia van Schalkwijk[2], Sabri Cebeci[2], Simon Jacobs[3], Wilma Wesselink[3], Guido Staring[3], Soenita Goerdayal[3], Andrei Prodan[2†], Ann Stijnman[3], Emma Teuling[3], Kerensa Broersen[3,4] and Herwig Bachmann[1,2]

[1]Systems Biology Lab, Vrije Universiteit Amsterdam, Amsterdam, The Netherlands

[2]Microbiology department, NIZO food research B.V., Ede, The Netherlands

[3]Food department, NIZO food research B.V., Ede, The Netherlands

[4]Applied Stem Cell Technologies, University of Twente, Technical Medical Centre, Enschede, The Netherlands

* Correspondence: Herwig Bachmann (Herwig.Bachmann@nizo.com)

#Present affiliation: CJ Research Centre Europe, Wageningen, The Netherlands

† Present affiliation: Single Cell Discoveries, Utrecht, The Netherlands

Supplementary Information S1: Measurement of volatiles and characterization of raw materials

Data normalization of GC-MS data (sterile unfermented plant proteins)

In the absence of internal standards, the obtained GC-MS data is subject to matrix effects which influences the response factor of a volatile compound. This complicates comparison between samples. Within our experiment, this matrix effect is caused by differences between plant proteins, dry matter content (5% for potato protein vs 10% for others), initial pH, and analysis batch. This can be seen through the range and distribution of volatile peak areas (Supplementary Figure 1). Despite lower dry matter content, potato protein showed an overall shift to a higher peak area resulting in an impression of high relative abundance of most volatiles.

Among detected volatiles, we observed that ethylacetate and 2-pentylfuran levels are consistently among the lowest and the highest, respectively. In the case of almond, one of the four replicates (Almond 3, removed from further analysis) seemed to be an outlier resulting in the larger standard deviations in the boxplots (Supplementary Figure 1). However, the presence of the outlier sample did not influence ethylacetate measurements and its standard deviation remained small despite being at the lower range of the data with generally a lower signal to noise ratio. For 2-pentylfuran, it is commonly reported as the product of lipid oxidation. While its presence is expected, its unusually high level among other furans (100- to 1000-fold higher) is likely to indicate high response factors in all matrices. Altogether, the presence of a consistent volatile species at minimum and maximum as well as overall shift in data distribution justifies scaling and centering of data to compare between samples. Scaling and centering for this blank (no incubation with microorganism) dataset was done within samples. For heatmap visualization, scaling and centering within volatiles was done (using the generic "scale" function in R). Averages of peak areas of detected volatiles are summarized in Table S1.

Common plant proteins shared the majority of detected volatiles

To understand similarities and differences between commercial protein isolates and concentrates, we performed an analysis on the volatiles detected in pea, almond, oat, and potato protein, representing legumes, stone fruit, cereals, and tubers, respectively. Based on normalized data (Supplementary Figure 2A), the relative composition of pea and oat proteins seems to be dominated by similar volatiles, while potato and almond proteins show distinctive volatile signatures. This is potentially attributed to differences in raw material and their processing (Supplementary Information S2). From a total of 42 volatiles measured across proteins, 27 of them were detected in all proteins and 7 of the remaining ones were detected in at least 3 proteins (Supplementary Figure 2B). These volatiles can be grouped based on their compound classes (Supplementary Figure 2C). Overall, the reported aldehydes, alcohols, ketones, and furans can be associated with lipid oxidation and/or Maillard reaction, and they are largely shared, albeit at different ratios.

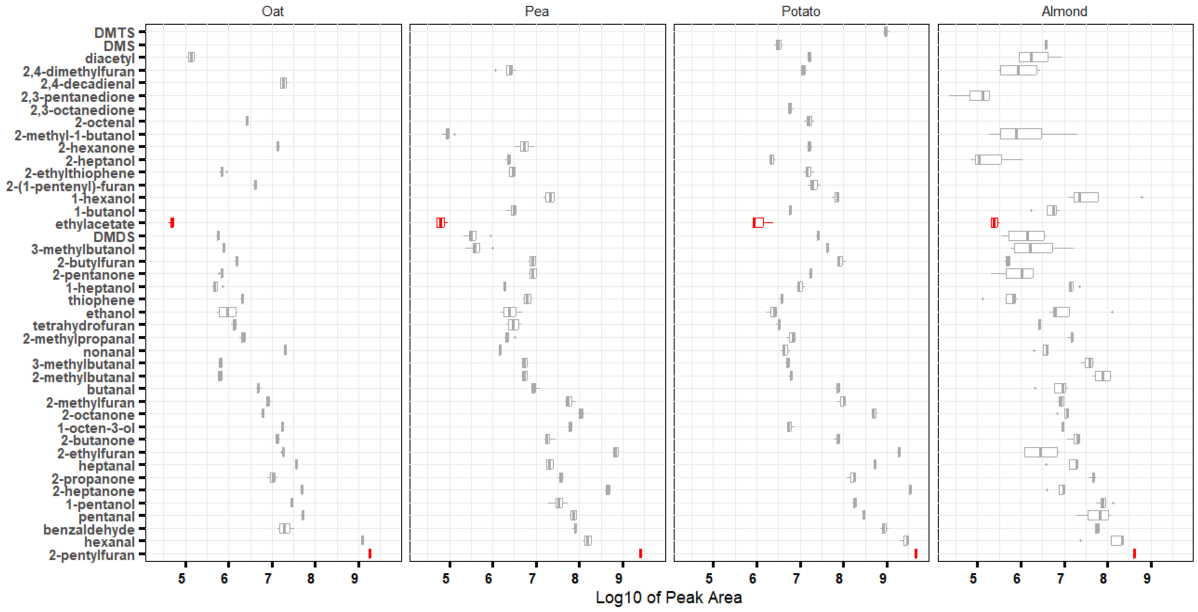

Supplementary Figure 1: Distribution of volatile peak areas (raw data) for each sterilized plant protein (n=3 for potato, n=4 for pea, almond and oat) shown as boxplots. Ethylacetate (consistently small peak area) and 2-pentylfuran (consistently large peak area) are depicted in red.

A commercial potato protein isolate used in this study shows overall low abundance of most detected volatiles (Supplementary Figure 2) with only a few volatiles including a few ketones, heptanal and its key compound dimethyl trisulfide (DMTS) ("cooked potato"[1,2]) found in higher abundance relative to other proteins. In contrast, almond protein concentrate displayed a high relative abundance of key compound benzaldehyde[3] and "green" aliphatic aldehydes (C5-C7), of which the latter are expected from an oleaginous protein concentrate. Despite purity differences in pea protein isolate and oat protein concentrate, they share 25 abundant volatiles. Additionally, 2,4-decadienal which is considered a less volatile compound, could only be detected in oat protein. While other proteins may contain 2,4-decadienal, their concentrations are likely too low to allow detection by headspace analysis. Furans seem to be abundant in all samples, which may be indicative of thermal degradation[4] and likely formed during heat treatments and/or sterilization of proteins. Aside of furans, aldehydes and ketones are consistently abundant in all four proteins and, consequently, are the target priority of off-flavor removal.

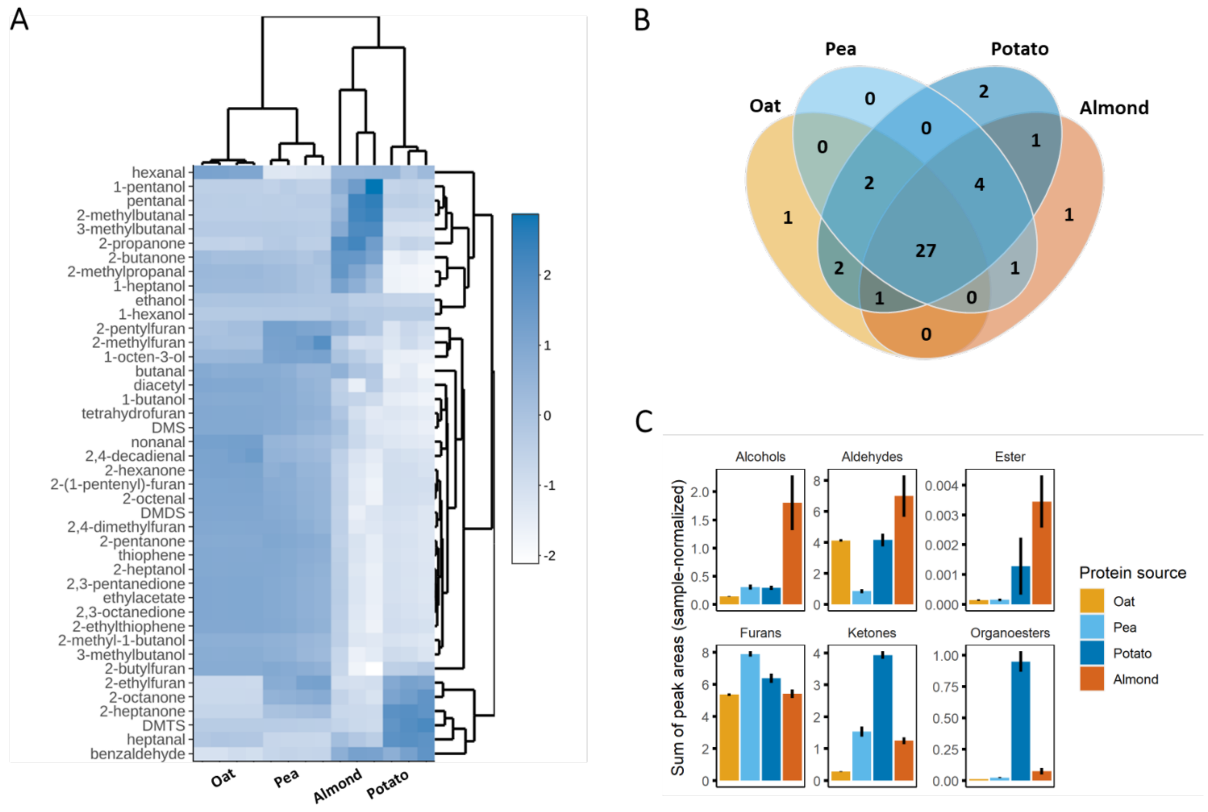

Supplementary Figure 2: Semi-quantitative headspace analysis of highly volatile compounds in potato, almond, pea, and oat protein. Panel A shows relative abundance of volatiles. Panel B shows a Venn diagram of detected volatiles. Panel C shows the sum peak areas per compound classes. Vertical error bars represent standard deviation from measurement replicates. N=3 for almond and potato proteins, n=4 for oat and pea proteins. Peak area values are sample-normalized to allow for comparison across matrices.

Table S1: GC-MS peak areas [log10 transformed] (unprocessed) of detected volatiles in sterile, unfermented plant proteins.The mean and standard deviation of 3 biological replicates are shown.

Class	Subclass	Substrate	Flavor[1]	Odor threshold (ppb in water)	Oat	Pea	Potato	Almond
Alcohols	unsaturated branched	1-octen-3-ol	Mushroom, earthy	1	7.24 ± 0.02	7.78 ± 0.05	6.76 ± 0.08	6.97 ± 0.02
	branched	2-methyl-1-butanol	Cocoa, whisky, leather-like		n.d.	4.96 ± 0.1	n.d.	6.1 ± 0.78
		3-methylbutanol	Fruity, banana, whiskey	250-300	5.89 ± 0.01	5.64 ± 0.23	7.64 ± 0.01	6.36 ± 0.59
	aliphatic (secondary)	2-heptanol	Fruity, citrus, green		n.d.	6.37 ± 0.05	6.36 ± 0.05	5.31 ± 0.53
	aliphatic	ethanol	Sharp, pungent	100000	5.97 ± 0.2	6.41 ± 0.2	6.37 ± 0.11	7.09 ± 0.59
		1-butanol	Fermented, fruity	500	n.d.	6.46 ± 0.09	6.76 ± 0.01	6.66 ± 0.26
		1-pentanol	Cereal, fruity, bread	4000	7.45 ± 0.01	7.52 ± 0.16	8.26 ± 0.04	7.91 ± 0.14
		1-hexanol	Green, herbal	2500	n.d.	7.31 ± 0.11	7.84 ± 0.07	7.66 ± 0.67
		1-heptanol	Green, fatty	3	5.71 ± 0.09	6.28 ± 0.01	7 ± 0.08	7.19 ± 0.1
Ester	aliphatic	ethylacetate	Sweet, chemical, nail varnish	5.0-5000.0	4.69 ± 0.05	4.81 ± 0.11	6.07 ± 0.22	5.4 ± 0.08
Organosulfur	branched heteroaromatic	2-ethylthiophene	Roast, coffee, meaty (maillard)	na	5.85 ± 0.06	6.45 ± 0.07	7.2 ± 0.1	n.d.
	heteroaromatic	thiophene	Garlic (Maillard)		6.31 ± 0.03	6.81 ± 0.09	6.57 ± 0.04	5.68 ± 0.32
	branched	DMS	Cooked vegetables	0.3-1	n.d.	n.d.	6.5 ± 0.08	6.57 ± 0.02
		DMDS	Sulfury, cabbage, onion	0.16-12	5.75 ± 0.02	5.57 ± 0.23	7.41 ± 0.02	6.12 ± 0.45
		DMTS	Sulfurous, onion	0.005-0.01	n.d.	n.d.	8.99 ± 0.05	n.d.
Ketones	diketones	diacetyl	Buttery, creamy	2.3-6.5	5.13 ± 0.08	n.d.	7.22 ± 0.04	6.34 ± 0.41
		2,3-pentanedione	Creamy, buttery, cheesy		n.d.	n.d.	n.d.	4.97 ± 0.38
		2,3-octanedione	Earthy, green dill		n.d.	n.d.	6.78 ± 0.06	n.d.
	furans	2-(1-pentenyl)-furan	roasted		6.6 ± 0.03	n.d.	7.32 ± 0.12	n.d.
		tetrahydrofuran	Used as solvent		6.14 ± 0.05	6.48 ± 0.14	6.5 ± 0.04	6.43 ± 0.03
		2,4-dimethylfuran	Meaty, roasted		n.d.	6.35 ± 0.18	7.08 ± 0.06	5.95 ± 0.44
		2-methylfuran	Chocolate like		6.92 ± 0.04	7.78 ± 0.1	7.96 ± 0.08	6.93 ± 0.06
		2-ethylfuran	Bready, malt, cocoa, nutty, coffee		7.26 ± 0.02	8.84 ± 0.06	9.28 ± 0.03	6.48 ± 0.39

[1] Leffingwell – Flavor Base

		2-butylfuran	Fruity, sweet, spicy		6.18 ± 0.02	6.92 ± 0.07	7.93 ± 0.08	5.7 ± 0.05
		2-pentylfuran	Fruity, green, earthy, waxy	6	9.25 ± 0.01	9.4 ± 0.01	9.68 ± 0.02	8.62 ± 0.02
	aliphatic	2-propanone	nail varnish remover, chemical	500000	7.02 ± 0.08	7.58 ± 0.04	8.19 ± 0.08	7.67 ± 0.06
		2-butanone	Like aceton, chemical fruity	50000	7.13 ± 0.04	7.28 ± 0.1	7.86 ± 0.06	7.27 ± 0.12
		2-pentanone	Sweet, fruity, banana	70000	5.83 ± 0.03	6.93 ± 0.08	7.24 ± 0.02	5.91 ± 0.4
		2-hexanone	Fruity, meaty, buttery		7.14 ± 0	6.73 ± 0.16	7.21 ± 0.06	n.d.
		2-heptanone	Cheese, fruity	140-3000	7.69 ± 0.02	8.66 ± 0.05	9.54 ± 0.01	6.89 ± 0.17
		2-octanone	Earthy, cheesy	50	6.79 ± 0.01	8.04 ± 0.05	8.72 ± 0.06	7.01 ± 0.1
Aldehydes	aromatic	benzaldehyde	almond	350	7.3 ± 0.15	7.9 ± 0.04	8.95 ± 0.08	7.77 ± 0.05
	unsaturated	2,4-decadienal	Fatty, oily, oxidised fat	0.07	7.27 ± 0.08	n.d.	n.d.	n.d.
		2-octenal	Fatty, green, citrus	3	6.41 ± 0.02	n.d.	7.21 ± 0.1	n.d.
	branched	2-methylpropanal	Floral, green, fresh aldehydic, chocolate	0.1-23	6.34 ± 0.06	6.36 ± 0.09	6.81 ± 0.09	7.17 ± 0.05
		3-methylbutanal	Chocolate, fruity, malty	0.2-2	5.81 ± 0.03	6.74 ± 0.07	6.71 ± 0.04	7.56 ± 0.11
		2-methylbutanal	Chocolate, fruity, malty	1	5.8 ± 0.04	6.74 ± 0.06	6.78 ± 0.03	7.88 ± 0.19
	aliphatic	butanal	Cocoa, musty, green	9-37.3	6.69 ± 0.05	6.96 ± 0.07	7.86 ± 0.04	6.84 ± 0.3
		pentanal	Fruity, berry, chocolate	12.00-42.00	7.71 ± 0.03	7.86 ± 0.07	8.47 ± 0.01	7.75 ± 0.32
		hexanal	Fresh, green, grass	4.5	9.09 ± 0.01	8.19 ± 0.09	9.42 ± 0.09	8.1 ± 0.42
		heptanal	Green, fatty	3	7.57 ± 0.02	7.32 ± 0.08	8.74 ± 0.02	7.12 ± 0.32
		nonanal	Waxy, fatty, orange peel, citrus, cucumber	1	7.31 ± 0.02	6.17 ± 0.02	6.65 ± 0.09	6.54 ± 0.13

Supplementary information S2: Differences in raw material and processing

Commercial potato protein used in this study shows overall low abundance of most detected volatiles (Figure 1A) with only 6 volatiles (2-heptanone, DMTS, heptanal, benzaldehyde, 2-octanone and 2-ethylfuran) found in higher abundance relative to other proteins. Among these volatiles, DMTS ("sulfurous", "onion") was particularly found only in potato protein and it contributed considerably to the total volatiles (Supplementary Figure 2C). The prominence of DMTS agrees with other studies where the compound was reported to be one of the main contributors to cooked potato aroma[5,6]. Aside from DMTS, the ketones of 2-heptanone ("cheese", "fruity") and 2-octanone ("cheese", "earthy") are other main contributors which further distinguish the volatile composition of potato proteins. Overall, potato protein is still a relatively clean matrix, and this can be associated with its high purity isolation using EBA (expanded bed adsorption) chromatography[7]. Also, a low-fat content might contribute to undetectable off-flavor precursors of unsaturated fatty acids (0.2% fat, Table S2).

In almond protein concentrate, higher amounts of benzaldehyde were observed. This is expected since benzaldehyde is a signature volatile that gives characteristics to almond aroma. Additionally, we observed a high contribution of both alcohols and aldehydes with "green" characteristics such as pentanal, pentanol, hexanal, hexanol, and heptanol. Additionally, all Strecker aldehydes (3-methylbutanal, 2-methylbutanal, and 2-methylpropanal) are higher in almond protein. This is in line with other reports[8] and expected from the higher abundance of branched chain amino acids in almond. The latter suggests Maillard reaction that occurs during processing. This protein concentrate is produced by partially defatting almond through mechanical press and subsequent thermal and pneumatical treatment. Around 10-11% unsaturated fat remains (*product specification sheet*), which is the highest among the other proteins and corresponds to the abundance of various "green" products of lipid oxidation.

In pea protein isolate and oat protein concentrate, 25 volatiles are shared as abundant volatiles despite purity differences. Furan volatiles are particularly more abundant in these proteins, accounting for more than 50% of total scaled volatiles. During processing, moisture was removed from oat and pea proteins using roller-drying and spray-drying, respectively. High formation of furans suggests that temperatures above 110°C were used during drying[9], allowing Maillard reactions to occur. It remains to be explained how furan formation occurs in pea protein isolate where sugars are barely present (Table S3). However, it was observed earlier that furan abundance increases at 30C storage of pea protein isolate, while its diversity increases with exposure to light[10]. In terms of lipid oxidation products, oat protein concentrate contains roughly 14% unsaturated fat, while pea protein isolate contains approximately 3.4%. This difference is reflected in the formation of green aldehydes, particularly heptanal and nonanal, which are found to be higher in oat protein concentrate.

As described above, plant protein concentrates and isolates typically contain a wide range of molecules. While we have no detailed data on this, it is commonly accepted that e.g. due to seasonal differences variations of the composition occur even between different batches of the same product which is a challenge for product development.

Table S2: General chemical analysis of plant proteins used in this study

**Protein Origin**	**Protein %**	**Carbohydrate %**	**Fibre %**	**Fat %**	**Moisture %**	**Note**	**Source**
Oat	52-56	20-24	2-3	16-18	3-6	Fat comprised of approximately 42% linoleic acid, 36% oleic acid, 16% palmitic acid, 2% a-linolenic acid, and 4% other fatty acids (C20-C24), carbohydrate in the form of maltodextrins and fibre as beta glucan.	[11]; Product specification sheet
Pea	84-88	0.7-0.8	1.4-2.4	4-9	3-7	Saturated fat is between 0.6 and 2.1	Product specification sheets
Almond	40-48.7	34-41	15-19	5.3-12	<=6	Unknown if almond was raw or blanched	[12]; Product specification sheet
Potato	90.5	<0.2	3.5	0.2			[13]

Supplementary information S3: Analysis of neutral mono, di-saccharide in plant protein

Table S3: High Performance Ligand Exchange Chromatography (HPLEC) detection of sugars in plant protein powders. Duplicate measurements are reported individually.

	Amount (mg/g)															
	Stachyose	Raffinose	Sucrose	Maltose	Lactose	Glucose	Lactulose	Xylose	Galactose	Rhamnose	Arabinose	Mannose	Fructose	Meso-Erythritol	Mannitol	Total
Pea	n.a.	n.a.	n.a.	n.a.	n.a.	n.a.	n.a.	n.a.	n.a.	n.a.	n.a.	n.a.	n.a.	n.a.	n.a.	0.0
	n.a.	n.a.	n.a.	n.a.	n.a.	n.a.	n.a.	n.a.	n.a.	n.a.	n.a.	n.a.	n.a.	n.a.	n.a.	0.0
Oat	n.a.	n.a.	5.94	n.a.	n.a.	n.a.	n.a.	n.a.	n.a.	n.a.	n.a.	n.a.	n.a.	n.a.	n.a.	5.9
	n.a.	n.a.	5.96	n.a.	n.a.	n.a.	n.a.	n.a.	n.a.	n.a.	n.a.	n.a.	n.a.	n.a.	n.a.	6.0
Potato	n.a.	n.a.	n.a.	n.a.	n.a.	n.a.	n.a.	n.a.	n.a.	n.a.	n.a.	n.a.	n.a.	n.a.	n.a.	0.0
	n.a.	n.a.	n.a.	n.a.	n.a.	n.a.	n.a.	n.a.	n.a.	n.a.	n.a.	n.a.	n.a.	n.a.	n.a.	0.0
Almond	4.08	10.42	93.92	n.a.	n.a.	n.a.	n.a.	n.a.	n.a.	n.a.	n.a.	6.10	n.a.	n.a.	n.a.	114.5
	4.16	10.4	93.98	n.a.	n.a.	n.a.	n.a.	n.a.	n.a.	n.a.	n.a.	6.15	n.a.	n.a.	n.a.	114.7

Supplementary information S4: Sugar addition and pre-culture conditions

The concept of biopurification is based on limited production of typical fermentation end products due to limited availability of sugars in the substrate.

To initially establish the role of sugar addition, an experiment was performed within a panel of diverse strains (n=30). At the end of 24- or 72-hour incubations of pea with LAB (n=15) or yeast (n=15) cells, respectively, volatiles were measured. Without added sugar volatile reductions were comparable to conditions with added sugar. Meanwhile, alpha diketones (diacetyl, 2,3-pentanedione) and ethylacetate were highly produced by LAB and yeasts, respectively, in the presence of added sugar. The formation of these compounds is typically not desired. The data indicates that limited sugar availability still allows for the reduction of unwanted compounds, especially with some particular strains, while at the same time it limits production of volatile metabolites. Both are desired to produce neutral tasting ingredients. Further screening was therefore focused on selecting strains with the ability to reduce volatile compounds in the absence of added sugar.

A 24h - LAB

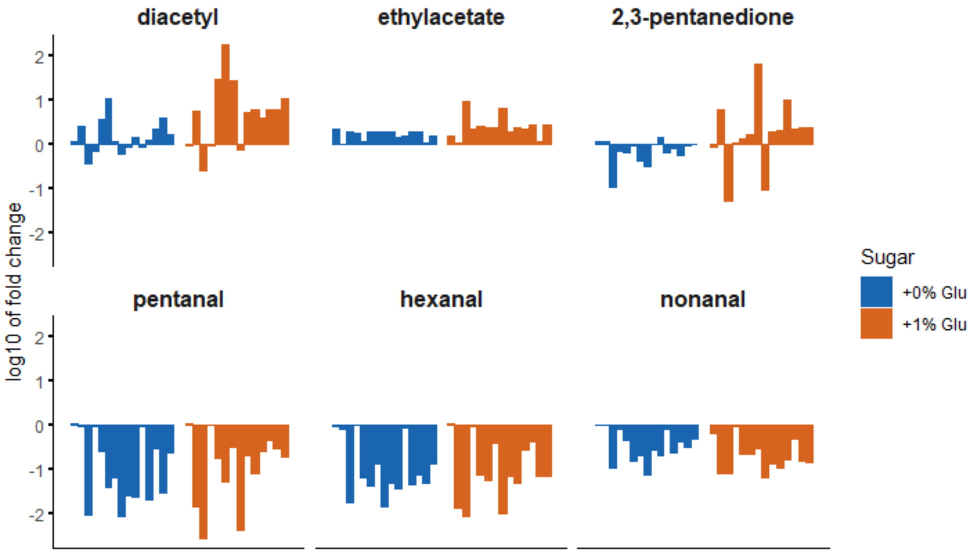

B 72h - yeasts

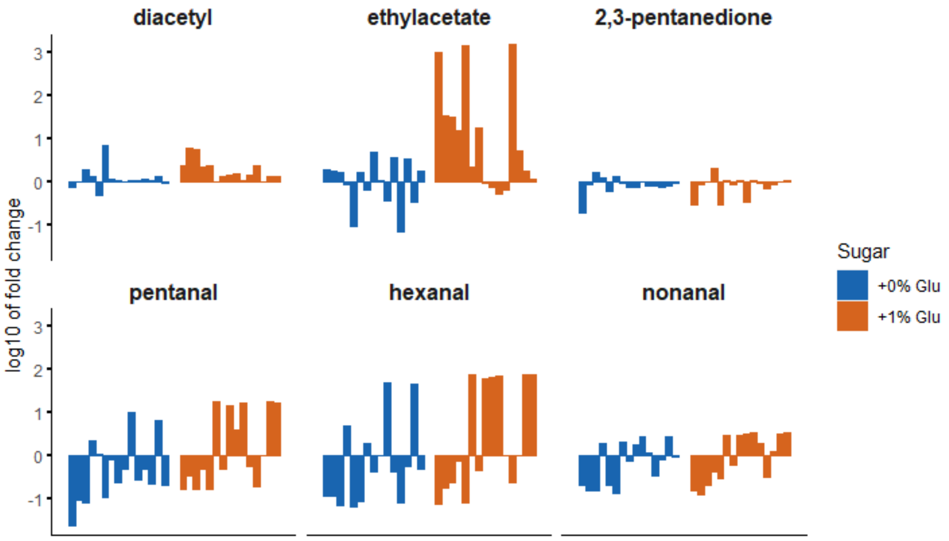

Supplementary Figure 3: Fold-change (Log10) of volatiles in biopurified pea samples compared to sterile unfermented samples. Protein solution was inoculated with 100-fold diluted early stationary cells and incubated for 24 hours or 72 hours at 30℃ for LAB (panel A) or yeast (panel B), respectively (each bar represents a different strain; n=1). When indicated, Glucose (1%) was added (bars from left to right correspond to each other for samples with and without glucose).

For the optimization of biopurification, strains that are already industrially used in food fermentations are attractive to apply. However, such strains might be homofermentative and therefore be low in the expression of alcohol dehydrogenase, resulting in limited removal of aliphatic aldehydes. Slow growth of facultative homofermentative organisms is associated with a shift towards heterofermentative metabolism, which is expected to come with higher ADH expression levels[14,15]. Such growth rates can be manipulated for instance by changing the type of sugar a strain is cultured on. To understand the effect of different sugars during culture preparation on the biopurification process, pea protein was inoculated with cells of the typically homofermentative strain *L. cremoris* MG1363[16] at roughly 1E8 cells/mL. Biopurification was performed either with or without the addition of sugar and after a 2-hour incubation period at 30 °C, volatiles were measured. The cells used were precultured with different sugars for a minimum of 15 generations, which allowed metabolic adaptation to different growth rates.

In the absence of added sugar in pea biopurification, volatile reduction was similar across sugar precultures. In the presence of added sugar, volatile reduction became more pronounced, particularly by cells cultured with sugar that led to low growth rates (< 0.4 /h). Up to 20-fold higher reduction of aldehydes in sugar-added pea was observed. However, the formation of pyruvate-derived

compounds such as ethylacetate, 2,3-butanedione, and 2,3-pentanedione concomitantly increased with the addition of sugar (Supplementary Figure 4).

In summary a growth rate reduction caused by using different carbon sources allowed to significantly increase the reduction of aldehydes during biopurification with a homofermentative organism.

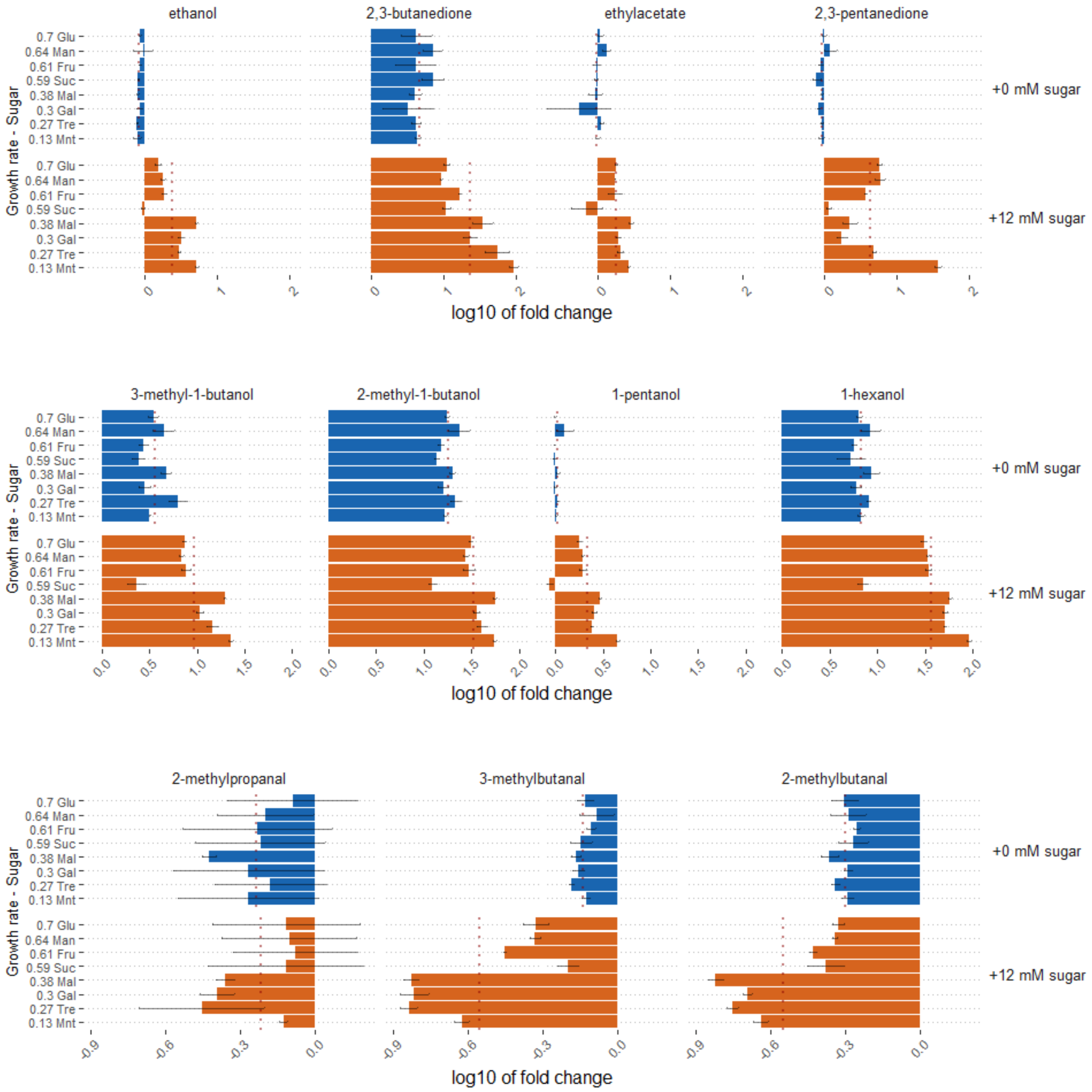

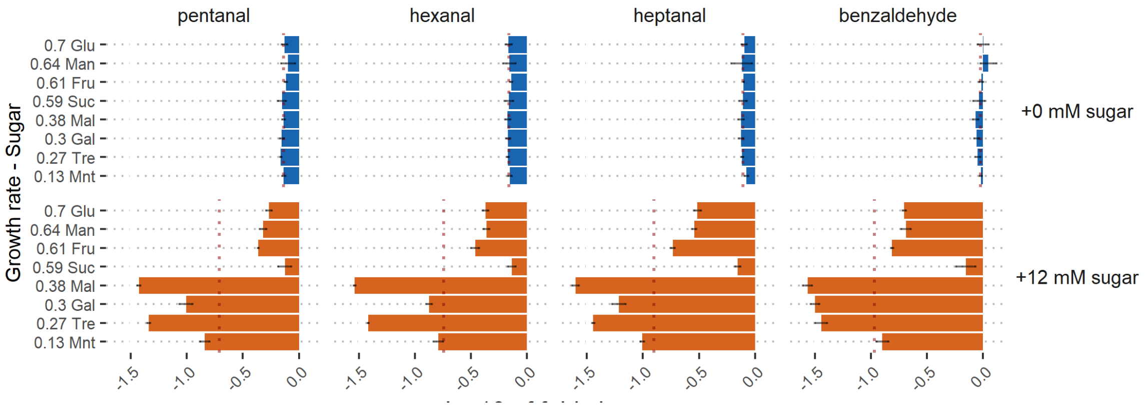

Supplementary Figure 4: Fold-change (Log10) of volatiles in biopurified pea samples in comparison to sterile unfermented samples. Biopurification was performed with roughly 1E8 cells/mL for 2 hours at 30℃. Cells were precultured for a minimum of 15 generations in sugars indicated on the left. Subsequently, cells were harvested at stationary phase, washed, and added to pea protein solution (10% dry matter) with or without the corresponding sugar added during preculture. Y-axis shows growth rate (/h) and 3-letter initial of sugar added during preculture: glucose (Glu), mannose (Man), fructose (Fru), sucrose (Suc), maltose (Mal), galactose (Gal), trehalose (Tre), and mannitol (Mnt). Error bars show standard deviation of the mean (n=3). The bottom panel is identical to Fig. 4 and shown here for completeness.

Supplementary information S5: Selection of biopurifying strains

An analysis of the heatmaps in Figure 2 (main text) (Supplementary Figure 5 to Supplementary Figure 8) shows that aldehydes and ketones were effectively reduced by obligate heterofermentative (*Lc. Carnosum* (n=4), *Lc. Fallax* (n=1), *Lc. Citreum* (n=1), *Lb. buchneri* (n=1), *Lb. parabuchneri* (n=1), and *Lb. brevis* (n=1)). Efficient reduction of aldehydes (but not ketones) was found in all strains of facultative heterofermentative *Lb. plantarum* (n=3) and obligate heterofermentative *Lc. Lactis* (n=3), *Lb. oris* (n=1), and *Lc. Suionicum* (n=2). Strain- and matrix-dependent variations in the efficiency or ability to degrade aldehydes and/or ketones were observed in, for instance, the obligate heterofermentative *Lc. Mesenteroides* (n=12), obligate homofermentative *Streptococcus thermophilus* (n=6), and facultative heterofermentative *Lb. curvatus* (n=5). Finally, *Lc. Gasicomitatum* and *Lc. Fructosum* deviated from the trend observed in most Leuconostocs by showing limited reduction of aldehydes. Both seem to be associated with either lower or non-functional ADH activity as a result of niche-specific evolution[17,18].

Based on our screening, strain rankings were calculated. For each volatile, log10(fold-change) was weighted by the volatile's sensory score (0,1,2,3,4 - Table S4; Table S5) and multiplied by -1. Higher scores indicate better biopurification. Fold change values were capped at 3 or -3 as the upper and lower limits. Values higher than 3 or lower than -3 were converted to 3 and -3, respectively. For each strain in each substrate, the weighted scores were averaged by chemical groups (e.g., aldehydes, ketones, etc). Subsequently, the average weighted scores per chemical group were added, which resulted in the strain's score in that substrate (Table S6). Total scores in all or individual substrates, combined with pH information were the bases for choices of individual experiments on e.g. techno-functional properties or biopurification optimizations.

The sensory scores were based on the ranking of qualitative descriptions (more or less offensive) as outlined in Table S4. Relevant thresholds or levels to which the concentration of volatiles eventually should be reduced depend on their ratios and the end application. For this reason, sensory evaluation of biopurification was tested in applications mimicking aqueous beverages and a semi-skim milk.

Table S4: Odour active compounds present in the sample which were perceived during GC-O and identified by GC-MS. The score in the first column indicates the potential impact of the volatile compound for off-flavor removal. A higher score indicates that a compound has a higher contribution to the off-flavor and therefore removal is more important. However, aroma perception will be strongly matrix dependent. Scores were estimated by a sensory expert based on known flavor defects as listed in the column description.

Score	Volatile	Description[2,3]
**4**	2,3-octanedione	Earthy, green dill
**4**	1-octen-3-ol	Mushroom, earthy
**4**	2-octanone	Earthy, cheesy
**4**	1-hexanol	Green, herbal
**4**	heptanal	Green, fatty
**4**	1-heptanol	Green, fatty

[2] Leffingwell – Flavor Base
[3] http://www.thegoodscentscompany.com/

**4**	2-pentylfuran	Fruity, green, earthy, waxy
**4**	2-octenal	Fatty, green, citrus
**4**	nonanal	Waxy, fatty, orange peel, citrus, cucumber
**4**	2,4-decadienal	Fatty, oily, oxidised fat
**4**	2-heptanol	Fruity, citrus, green
**4**	DMS	Cooked vegetables
**4**	thiophene	Garlic (Maillard)
**4**	DMDS	Sulfury, cabbage, onion
**4**	DMTS	Sulfurous, onion
**3**	hexanal	Fresh, green, grass
**2**	benzaldehyde	almond
**2**	2-ethylthiophene	Roast, coffee, meaty (maillard)
**2**	2-hexanone	Fruity, meaty, buttery
**2**	2-heptanone	Cheese, fruity
**2**	2,4-dimethylfuran	Meaty, roasted
**2**	2-(1-pentenyl)-	roasted
**1**	2-methylpropanal	Floral, green, fresh aldehydic, chocolate (Strecker aldehyde of valine)
**1**	butanal	Cocoa, musty, green
**1**	2-methylfuran	Chocolate like
**1**	3-methylbutanal	Chocolate, fruity, malty (Strecker aldehyde of leu)
**1**	2-methylbutanal	Chocolate, fruity, malty (Strecker aldehyde of ile)
**1**	2-ethylfuran	Bready, malt, cocoa, nutty, coffee
**1**	2-methyl-1-	Cocoa, whisky, leather-like
**1**	diacetyl	Buttery, creamy
**1**	2,3-pentanedione	Creamy, buttery, cheesy
**1**	ethylacetate	Sweet, chemical, also used as solvent in nail varnish
**1**	1-butanol	Fermented, fruity
**1**	2-pentanone	Sweet, fruity, banana
**1**	3-methylbutanol	Fruity, banana, whiskey
**1**	1-pentanol	Cereal, fruity, bread
**1**	2-butylfuran	Fruity, sweet, spicy
**1**	pentanal	Fruity, berry, chocolate
**0**	ethanol	Sharp, pungent
**0**	2-propanone	Associated with nail varnish remover, chemical
**0**	2-butanone	Like aceton, chemical fruity
**0**	tetrahydrofuran	Used as solvent

Table S5: GC-Olfactometry (GC-O) results reflecting the aroma active compounds after biopurification. Compounds are listed in the order of eluting from the GC.

	Description GC-O	Compound identified by MS	Description[4,5]
**1**	Sewage, sulfur, potato	Sulfur dioxide	Burnt matches, rotten egg
**2**	Sewage	Methanethiol	Sulfur, gasoline, garlic
**3**	Butter, dairy	Diacetyl	Butter
**4**	Chocolate, brown, unpleasant smell	3-methylbutanal	Ethereal aldehydic chocolate peach f atty
**5**	Garlic, glue, unpleasant	1-penten-3-ol	ethereal horseradish green radish chrysanthemum vegetable tropical fruity
**6**	Chocolate, sweet, butter	2,3-pentanedione	pungent sweet buttery creamy, caramellic, nutty cheesy
**7**	Green, old woolen coat, fresh	3-methyl-2-butanol	musty alcoholic fusel vegetable cide, cocoa cheesy, mild green fusel oil, fermented
**8**	Old wollen coat, cacao	3-methyl-1-butanol	musty alcoholic fusel vegetable cider , cocoa, cheesy
**9**	Green, earthy, apple, candy, musty	Hexanal	green fatty leafy vegetable fruity cle an woody apple
**10**	Brown, roast, sweet	Furfural	sweet woody almond bread baked
**11**	Sweet, fruity	Tr-2-hexenal	sweet almond bitter almond fruity green leafy apple plum , vegetable
**12**	Fresh, green, musty	1-hexanol	pungent ethereal fusel oily fruity alc oholic sweet green
**13**	Potato, methional	Methional	musty potato tomato earthy vegetab le, creamy
**14**	Green, sweet, fatty	2-heptenal (E-)	green fatty
**15**	Mushroom	1-octen-3-ol	mushroom earthy green oily fungal r aw chicken
**16**	Sulfur, plant waste	dimethyl-trisulfide	sulfurous alliaceous cooked savory m eaty eggy vegetable fresh onion green onion
**17**	Green, fatty, fresh	Octanal	aldehydic waxy citrus orange, green, peely
**18**	Coconut, fresh	2-ethyl-1-hexanol	citrus fresh floral oily sweet

[4] Leffingwell – Flavor Base
[5] http://www.thegoodscentscompany.com/

**19**	Mushroom, sweet	3-octen-2-one	earthy spicy herbal sweet mushroom hay blueberry
**20**	Cucumber, sour, old woollen coat, fatty	2-Octenal, (E)-	fatty green herbal
**21**	Mushroom, sulfur, dirty	3,5-octadien-2-one (E,Z)	fruity fatty mushroom
**22**	Mushroom, sulfur, pea, sour, liquorice, aniseed	1-octanol	waxy green,<br>orange, aldehydic, rose, mushroom
**23**	Green, fatty	Nonanal	waxy aldehydic citrus fresh green le mon peel cucumber fatty
**24**	Brown, smokey	2-methoxy-3-isopropylpyrazine	pea green<br>pea earthy beany chocolate nutty
**25**	Green, smells unpleasant, cucumber	2,6-nonadienal (E,Z)	green fatty dry cucumber violet leaf
**26**	Brown, green, pyrazine	2-methoxy-3-sec-butylpyrazine	musty pea green<br>pea galbanum pepper bell pepper
**27**	Green, cucumber	2-nonenal	fatty green waxy cucumber melon
**28**	Musty, green, pyrazine	3-Isobutyl-2-methoxypyrazine	pea green pea pepper bell pepper green galbanum
**29**	Fatty, green	2,4-Nonadienal, (E,E)-	fatty green cucumber fruit tropical fruit
**30**	Sweet, fresh, light, sulfur	2-Decenal, (E)-	fatty orange rose aldehydic floral gre en
**31**	Smokey, fresh, tea, sweet	1-decanol	fatty waxy floral orange sweet clean<br><br>watery
**32**	Fatty, fruity, fresh	2,4-Decadienal, (E,Z)-	orange sweet fresh citrus fatty green
**33**	Fatty, old shop, sweet	2,4-Decadienal, (E,E)-	orange sweet fresh citrus fatty green
**34**	Eugenol, lavender, almond, cinnamon, spice-like, burnt	Eugenol	tea herbal green wet tobacco leaf metallic woody spicy
**35**	Fatty, nutty, coconut	Gamma-undecalactone	fruity peach creamy fatty lactonic, a pricot ketonic, coconut
**36**	Ginger, sweet, green	Dodecanal	Soapy, waxy, aldehydic, citrus, gree n,<br><br>Floral, orange rind
**37**	Vanilla, coumarin,	Vanillin	sweet vanilla, creamy, chocolate

Table S6: Top 10 LAB and yeast strains, weighted score of volatile neutralization, and final pH in plant proteins. The pH of sterile, unfermented solutions are: 6.4 (almond and oat), 7.2 (pea), and 6.0 (potato).

Strain	Rank	Species	Weighted score					pH			
			Almond	Oat	Pea	Potato	Sum	Almond	Oat	Pea	Potato
LAB 98	1	*Lactobacillus buchneri*	7.98	12.42	3.73	2.40	26.54	5.54	5.77	7.05	5.79
LAB 77	2	*Lactobacillus parabuchneri*	9.00	9.61	3.85	3.46	25.92	5.53	5.85	7.06	5.90
LAB 70	3	*Lactobacillus brevis*	7.72	10.85	3.15	4.17	25.88	6.41	6.11	6.96	5.66
LAB 87	4	*Leuconostoc citreum*	7.15	14.94	3.21	-0.30	24.99	4.60	5.66	6.94	5.75
LAB 96	5	*Leuconostoc mesenteroides dextranicum*	10.35	10.46	4.56	-0.97	24.39	4.73	5.70	6.94	5.69
LAB 95	6	*Leuconostoc mesenteroides dextranicum*	10.39	9.91	4.29	-0.40	24.19	4.87	5.64	6.98	5.70
LAB 97	7	*Leuconostoc pseudomesenteroides*	6.99	11.57	4.59	0.98	24.12	4.73	5.66	6.93	5.65
LAB 69	8	*Leuconostoc mesenteroides*	9.52	10.11	2.57	1.02	23.23	4.49	6.65	7.01	5.48
LAB 61	9	*Leuconostoc mesenteroides mesenteroides*	5.71	9.88	8.33	-0.71	23.21	4.63	5.62	6.99	5.64
LAB 66	10	*Streptococcus thermophilus*	8.60	7.34	4.95	0.79	21.68	5.95	5.99	6.98	5.74
Yeast 33	1	*Pichia deserticola*	12.90	15.86	13.16	7.03	48.96	6.39	6.38	7.14	5.93
Yeast 20	2	*Pichia manshurica*	14.50	11.94	10.14	9.21	45.79	6.40	6.40	7.20	5.97
Yeast 37	3	*Barnettozyma californica*	14.08	10.45	9.85	11.06	45.44	6.20	6.28	7.08	5.99
Yeast 31	4	*Pichia scutulata*	13.77	7.72	9.96	5.98	37.42	6.36	6.41	7.20	5.97
Yeast 24	5	*Candida sake*	10.30	10.13	5.21	6.76	32.40	5.88	6.15	7.12	5.99
Yeast 30	6	*Pichia spartinae*	3.38	7.18	10.66	10.14	31.36	5.92	6.10	7.05	5.95
Yeast 34	7	*Wickerhamomyces subpelliculosus*	13.17	1.51	8.79	6.79	30.26	5.73	6.02	7.08	5.92
Yeast 17	8	*Pichia kluyveri*	11.52	6.15	6.54	3.44	27.65	6.46	6.35	7.13	5.98
Yeast 22	9	*Torulaspora microellipsoides*	6.01	6.59	4.22	7.87	24.69	5.66	6.05	7.07	5.97
Yeast 28	10	*Zygosaccharomyces bailii var. bailii*	8.83	5.67	5.28	4.40	24.19	4.47	4.93	6.86	5.91

Supplementary Information S6: Volatile profiles of biopurified protein

(extended Figure 2)

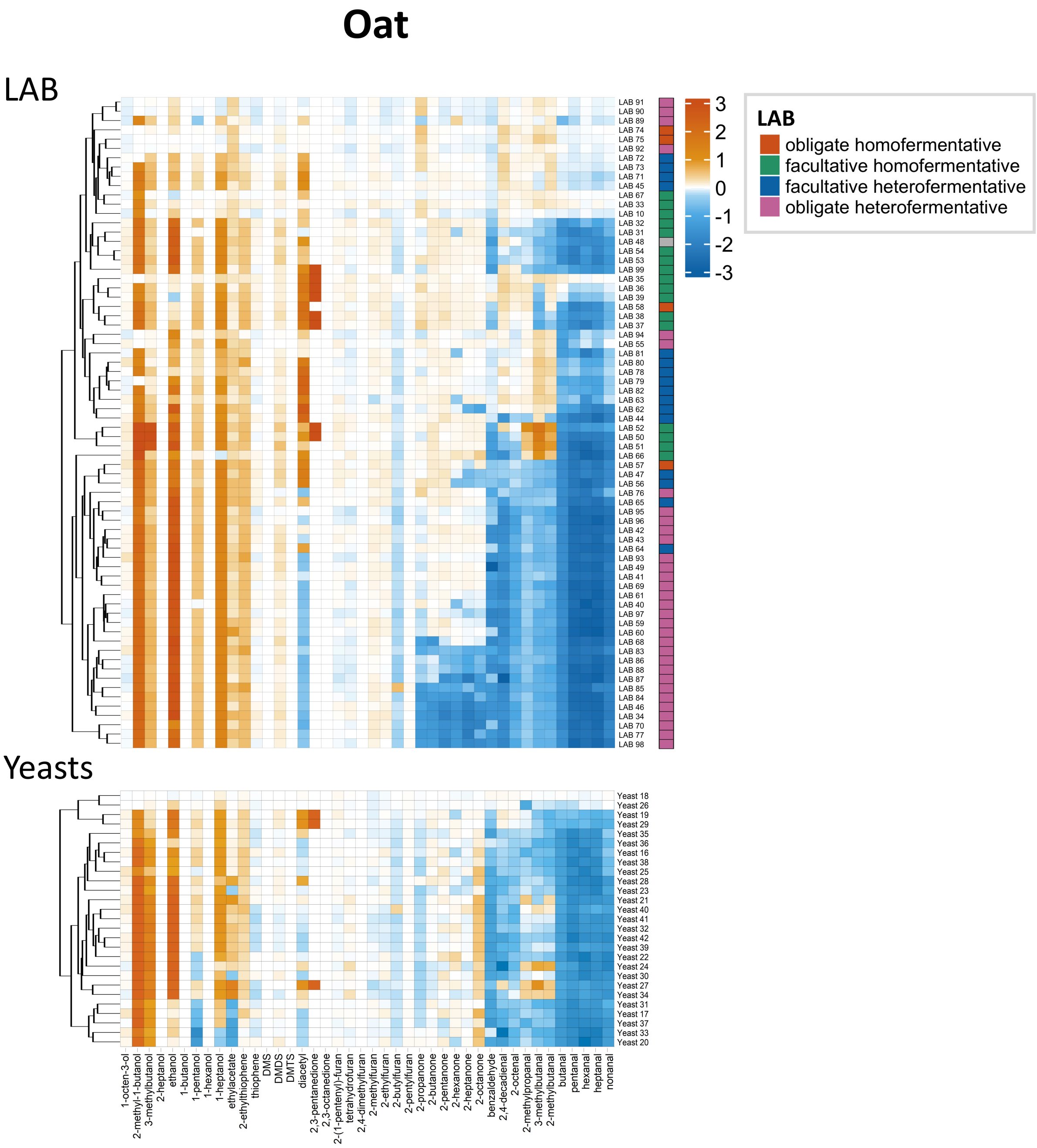

Supplementary Figure 5: Heatmap of log10-fold change of volatiles in oat protein. Oat protein solution (10% DM) was incubated for 24 hours with cells at an inoculation density of 10% of harvested and washed culture (early stationary)(n=1). Sterile and unfermented samples were used as reference.

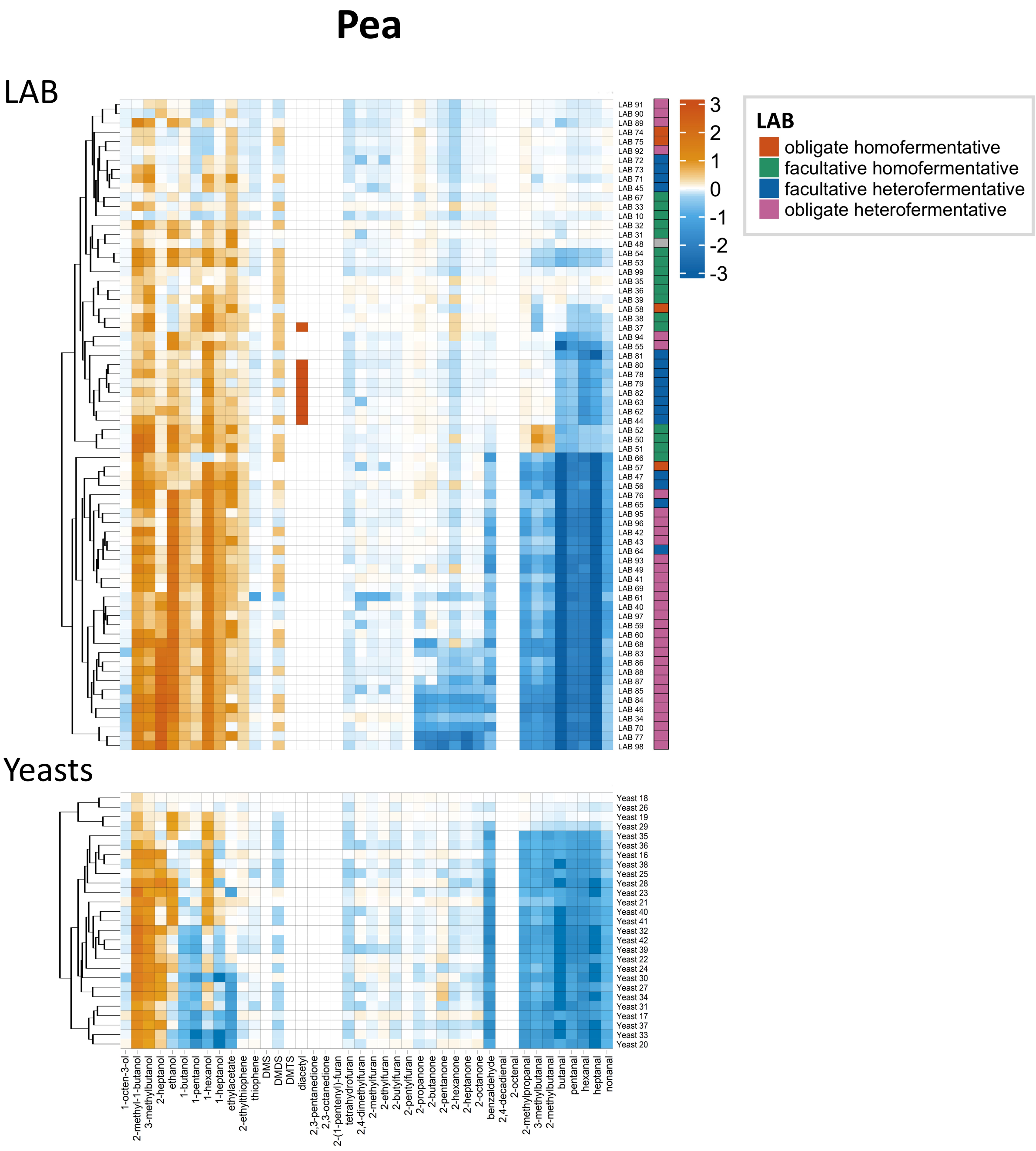

Supplementary Figure 6: Heatmap of log10-fold change of volatiles in pea protein. Pea protein solution (10% DM) was incubated for 24 hours with cells at an inoculation density of 10% of harvested and washed culture (early stationary)(n=1). Sterile and unfermented samples were used as reference.

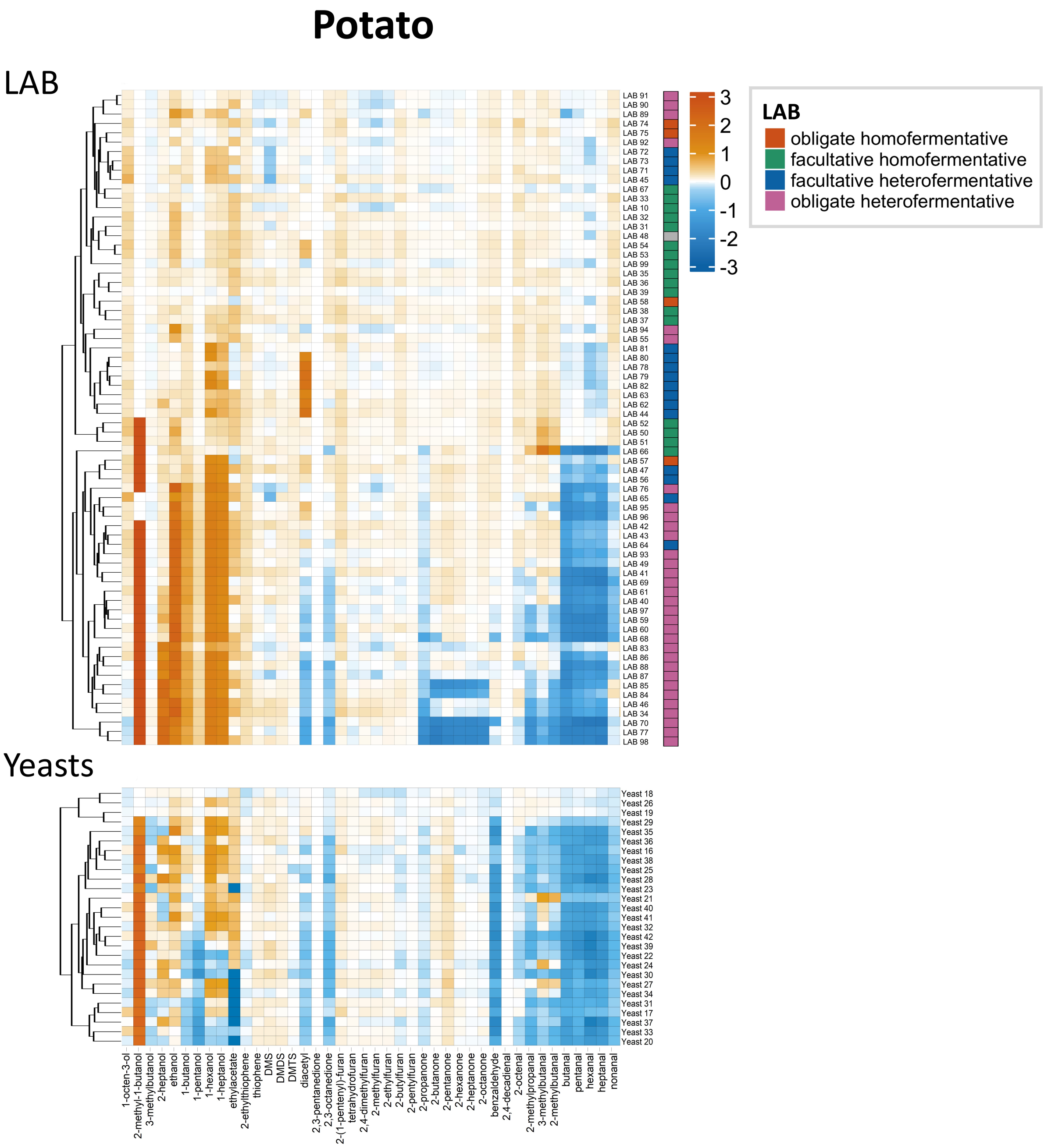

Supplementary Figure 7: Heatmap of log10-fold change of volatiles in potato protein. Potato protein solution (5% DM) was incubated for 24 hours with cells at an inoculation density of 10% of harvested and washed culture (early stationary)(n=1). Sterile and unfermented samples were used as reference.

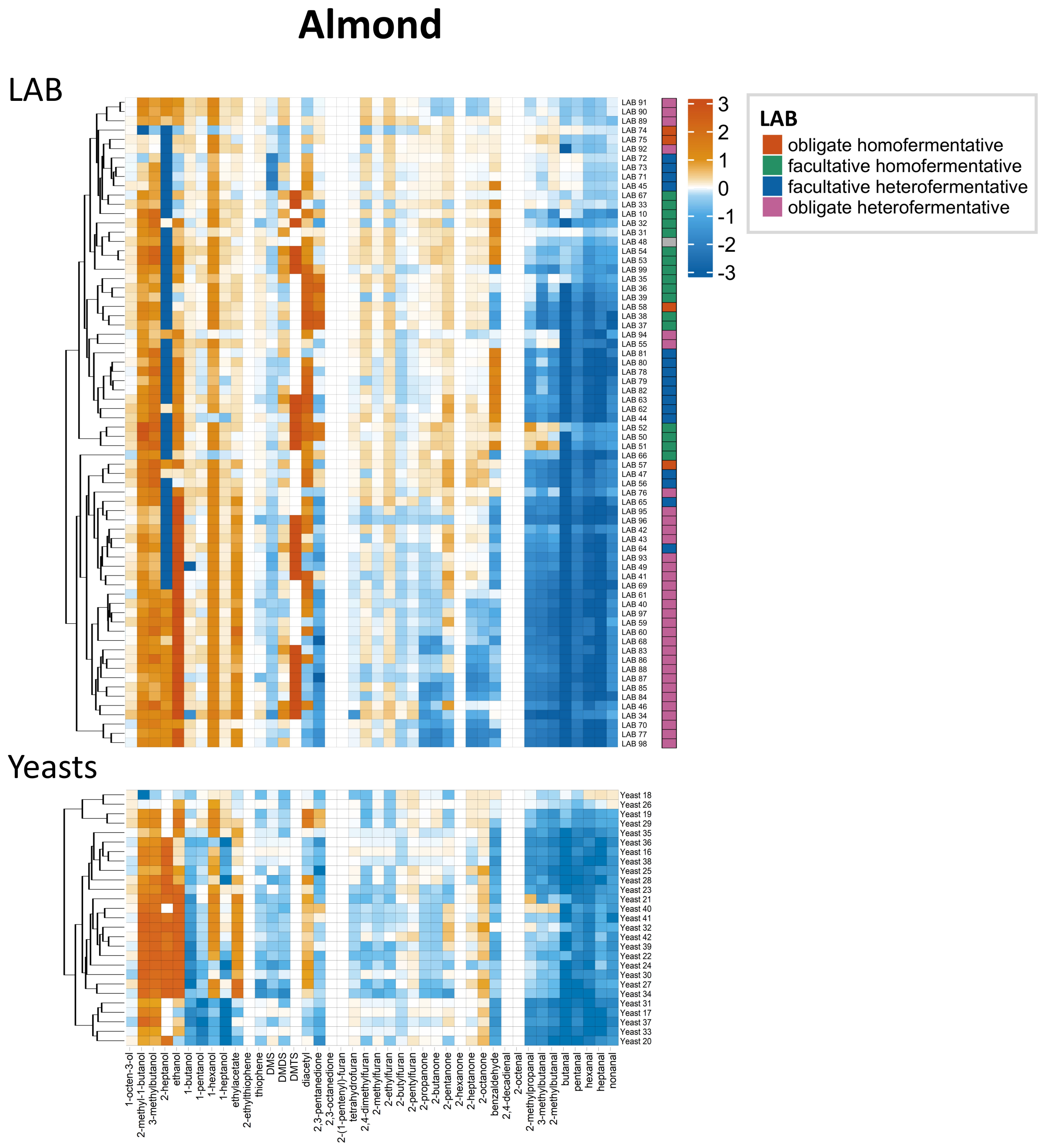

Supplementary Figure 8: Heatmap of log10-fold change of volatiles in almond protein. Almond protein solution (10% DM) was incubated for 24 hours with cells at an inoculation density of 10% of harvested and washed culture (early stationary)(n=1). Sterile and unfermented samples were used as reference.

Supplementary Information S7: Longer incubation and extra addition of NADH did not lead to further volatile reduction.

(Extended Figure 5)

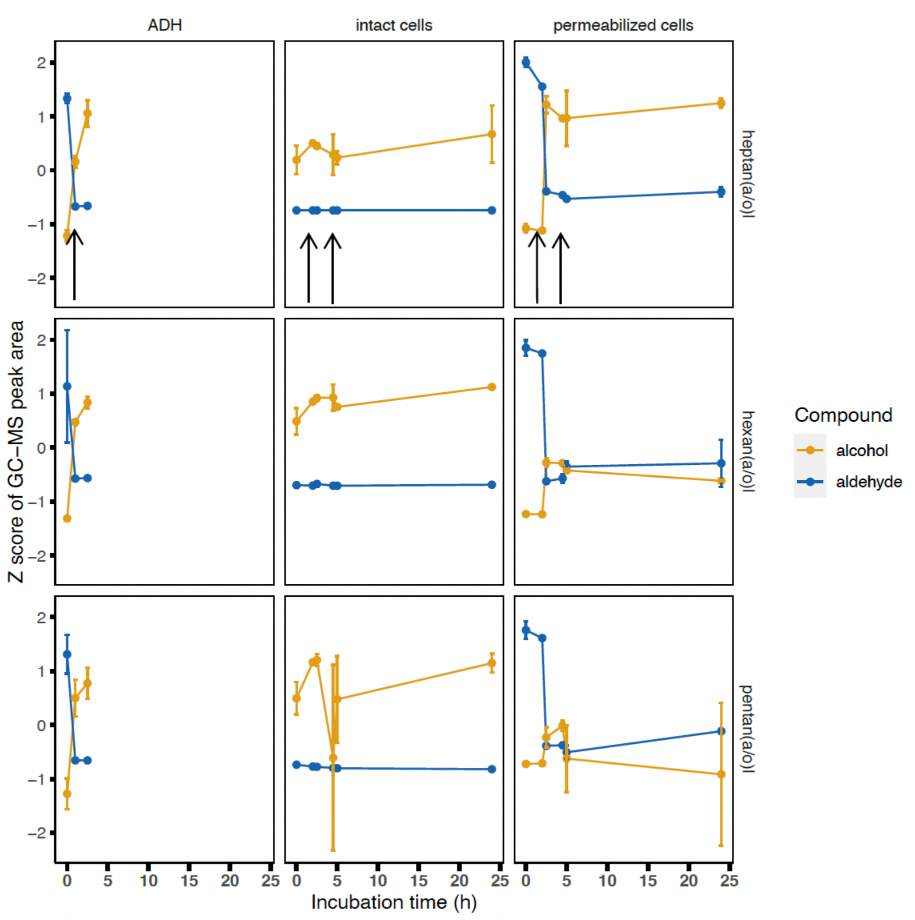

Supplementary Figure 9: Reduction of aldehydes to their corresponding alcohols with additional addition of NADH. Isolated ADH, intact LAB cells, and permeabilized LAB cells (columns from left to right) were used as biopurifying agent. Error bars represents standard deviation of (biological) replicates, (n=3 for ADH and n=2 for cells). NADH addition is indicated by back arrows in the first row. During sample preparation or NADH addition, samples were temporarily on ice before samples were frozen for later analysis. For ADH, the aliquot was immediately frozen at -20 ℃. In all conditions the majority of the aldehyde reduction occurred while the samples were on ice and before freezing. A second dose of NADH did not result in more reduction of aldehydes.

Supplementary Information S8: Optimization of biopurification - dry matter content, inoculation density and incubation times

Biopurification attempts in the presented study were mainly performed in aqueous setup at a dry matter content between 5 and 10% of plant proteins. Such setup was selected for experimental investigations due to practicality reasons. However, additional drying processes will be required for specific industrial applications, which requires additional energy. To investigate whether solid state biopurification is a manufacturing option, hydrated pea protein at 40% dry matter content was incubated with different densities of LAB61 (*Leuconostoc mesenteroides*). Such solid state biopurification was monitored for 24 hours at 30°C. Figures S9 and S10 show that the degree of aldehyde reduction was almost complete at the first measurement (<30 minutes after inoculation) when 1E8 cell/ml or more were used, and it took up to 24 hours when the inoculation density was decreased to 1E6 cells/ml. Overall the conversion of green aldehydes to their less potent alcohol derivatives is remarkably rapid. The colony forming units per gram of biopurified product reached similar levels for all inoculation densities after 24 hours (Supplementary Figure 12 – top panel), while at the same time the pH changes are minimal. The latter is expected due to the limited carbon sources available (Supplementary Figure 12 – lower panel). Together the data demonstrates that in the first hours of incubation with high inoculation densities volatile compounds can be quickly degraded while at the same time there is no detectable microbial growth or acidification.

Aldehydes decreased with time/cell density

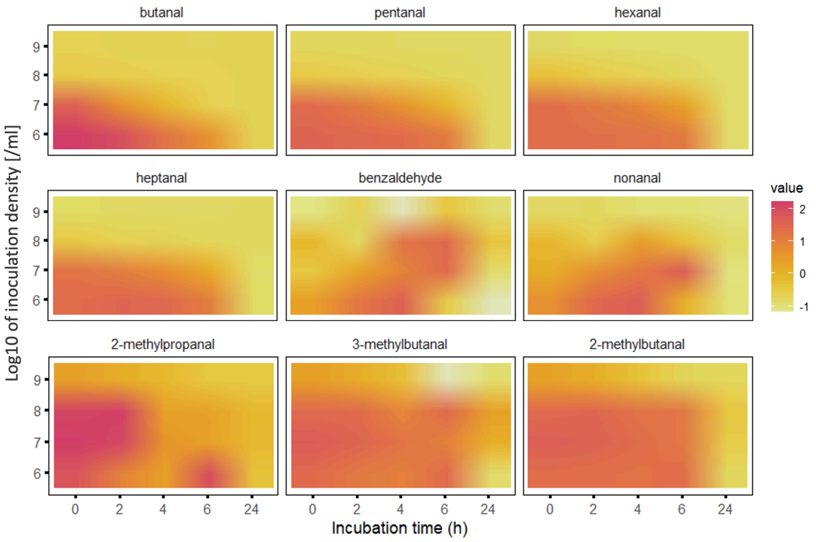

Alcohols increased with time/cell density

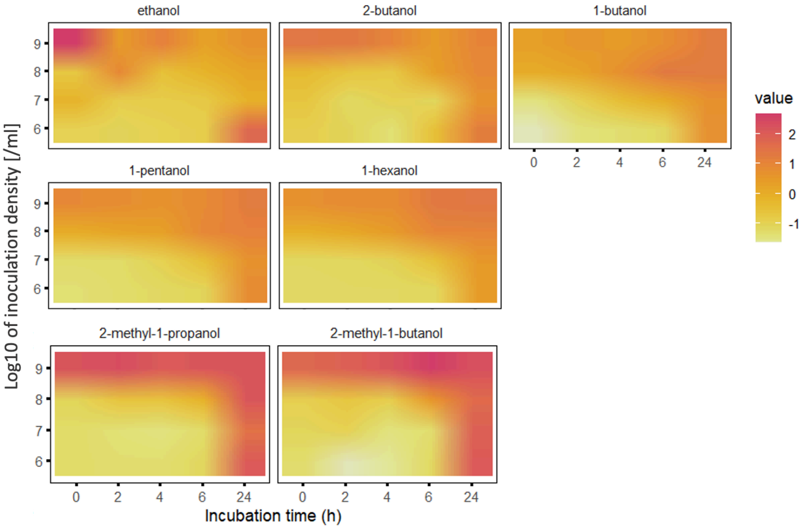

Supplementary Figure 10: Decrease of aldehydes and increase of alcohols as a function of inoculated cell density and incubation time during solid state biopurification (40% DM pea protein). Heatmaps are based on single measurements for each combination of incubation density and incubation time.

Pyruvate-derived compounds increased with time/cell density

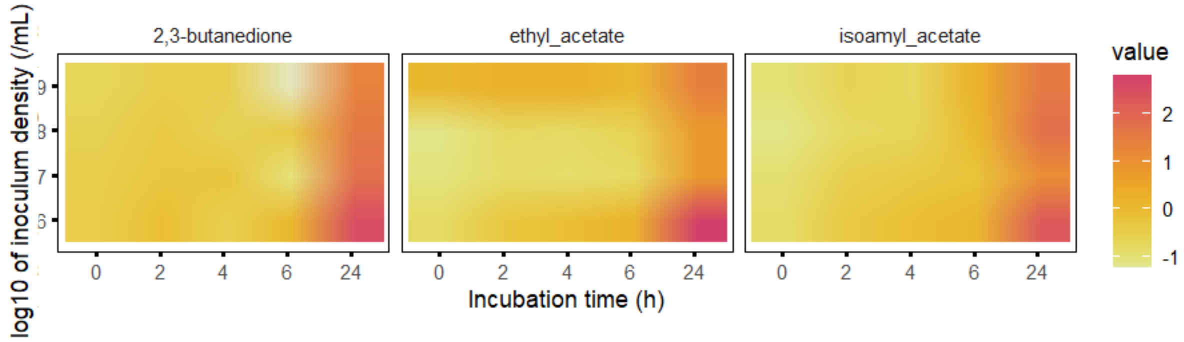

Alpha-diketones decreased with time/cell density

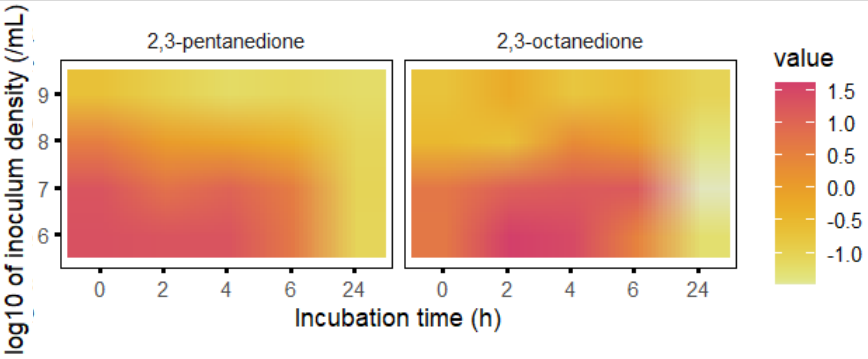

Supplementary Figure 11: Changes in volatiles as a function of inoculated cell density and incubation time during solid state biopurification (40% DM pea protein). (Upper panel) pyruvate-derived compounds mostly increased with time, which suggests coupling of metabolite formation to growth. (Lower panel) Non-diacetyl alpha diketones decreased with time and cell density. Heatmaps are based on single measurements for each combination of incubation density and incubation time.

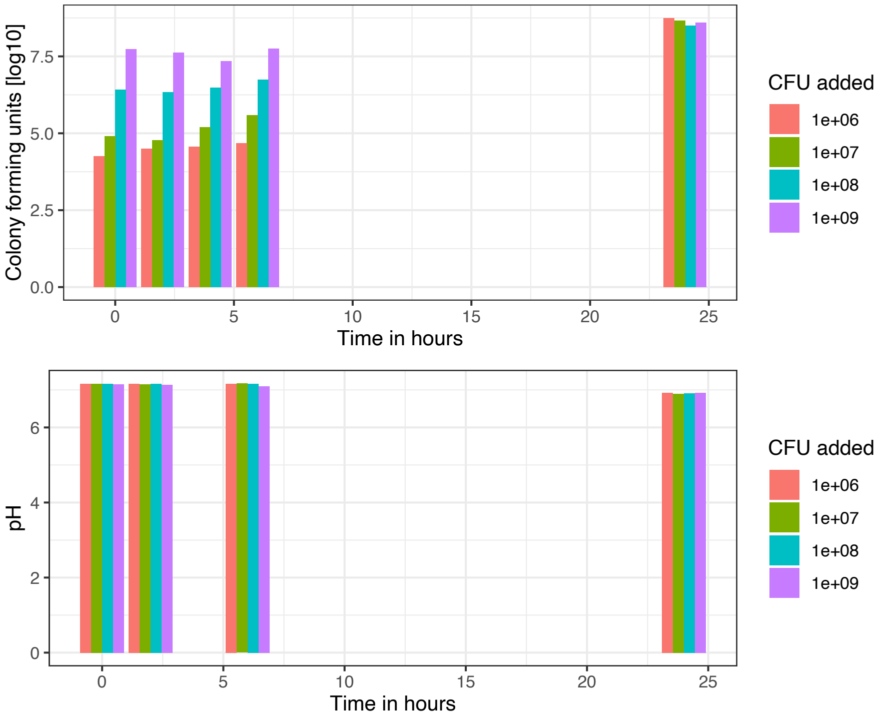

Supplementary Figure 12: Colony forming Unit determination (n=1) shows that dilution and plating of 40% DM pea protein results in a lower CFU count than what was added based on an OD measurement. This could be due to adhesion of cells to non-dissolved proteins or other matrix effects on plate counts. However, the data shows that CFU counts hardly change during the first 5 hours of incubation. After 24 hours of incubation the CFU counts of all samples increased to roughly 1E8 cells/gram of protein mixture (top panel). Similarly, the pH did not change in the first 6 hours at any inoculation density. After 24 hours of incubation a slight decrease of roughly 0.1 pH units is seen.

Supplementary Information S9: Sensory analysis, GC-Olfactometry

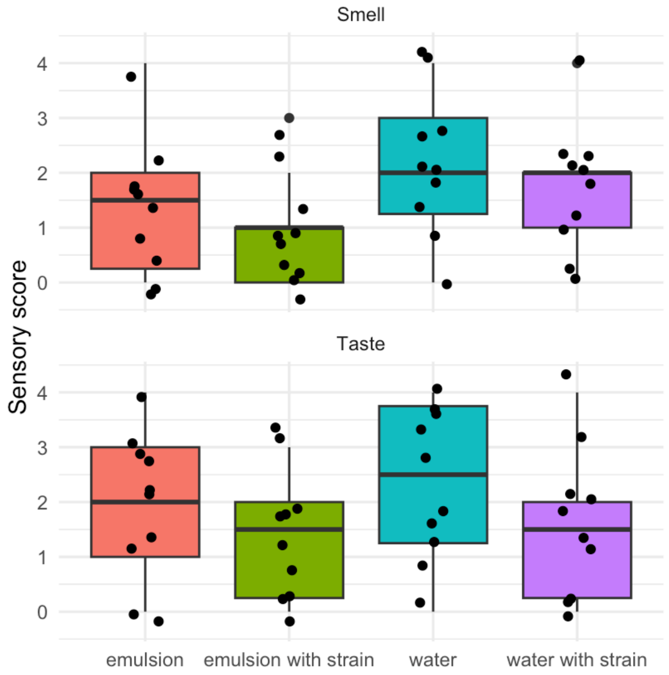

Supplementary Figure 13: CATA analysis showed a reduction of the pea attribute if biopurification (with strain) was applied to a pea protein suspension in water or a pea protein-based emulsion. Boxplots show the median and interquartile distances. Black dots show the individual datapoints of the 10 panel members.

For the sensory analysis of pea protein fermented with *L. buchneri* (LAB 98) flavor active volatile compounds were determined with GC-olfactometry (Table S5). The odour of the compounds identified ranged from sulfury (sulfur dioxide, methanethiol, methional, dimethyltrisulfide), fresh, green, grassy (hexanal, hexanol), creamy buttery, cheesy (diacetyl, 2,3-pentanedione, heptanone-2), cocoa, malty (3-methylbutanal), fatty, green, cucumber (octanal, nonanal, 2-heptenal, 2-octenal), mushroom (octen-3-ol, 1-octanol, 3,5-octadien-2-one), sweet vanilla (vanillin), sweet roast (furfural), pea, green (methoxypyrazines), fatty, coconut (gamma-undecalactone). Compounds such as methoxy substituted pyrazines, well-known for exhibiting the typical pea-like, green, beany and earthy aromas such as 2-methoxy-3-isobutylpyrazine and 2-isopropyl-3-methoxypyrazine[19–21] were also identified in this study by GC-O but not with GC-MS. This is due to the fact that these compounds have a very low sensory threshold (ppb level) but are not easily detected by MS or FID and can often only be detected by e.g. GC-O. Most of the odours identified in this study are considered unpleasant. Fermentation was shown to reduce (odour active) compounds belonging to the aldehyde and ketone classes by GC-MS. It is assumed that the aldehydes were reduced to the corresponding alcohols by the *Lactobacillus buchneri* which is known to possess reductase activity[22]. This is substantiated by the fact that an increase in the corresponding alcohols was observed by GC-MS (Supplementary Figure 14). In general, the odour threshold values for aldehydes in water are lower than the

corresponding alcohols resulting in a less intense off-flavour and (slight) change in odour character which will reduce the overall flavour intensity. The GC-O analysis of biopurified samples points to compounds that could be targeted in future experiments with e.g. mixed microbial cultures.

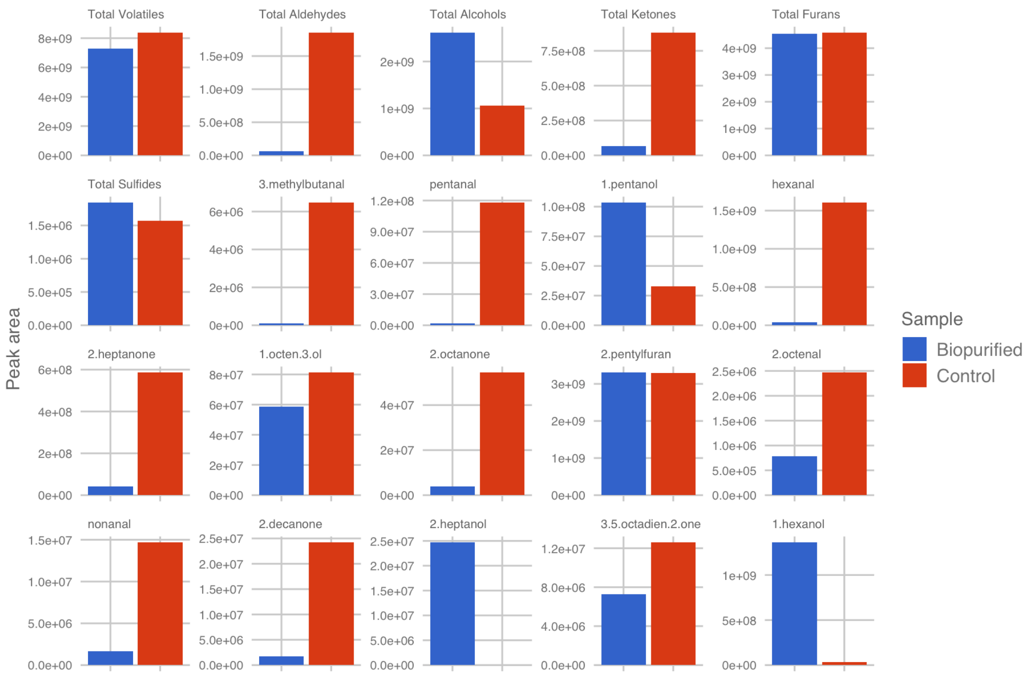

Supplementary Figure 14:GC-MS results of samples that were used for sensory analysis. Panels at the top indicate compound groups, whereas the bottom panels display selected volatiles from that data with biopurification (blue) and without (red). Measurements are based on individual samples that were offered to panel members.

Supplementary Information S10: Peptide and amino acid analysis of biopurified and *in vitro* digested plant proteins

To investigate the role of biopurification on protein degradation and subsequent in vitro digestibility we biopurified pea protein with four different microorganisms. The organisms were chosen to be proteolytic positive (*L. lactis* NCDO712), a yeast, and a lactic acid bacterium that performed well in terms of volatile removal (LAB61 – *Leuconostoc mesenteroides*; Yeast 20 - *Pichia manshurica*) and a species regularly studied in probiotic applications (LAB56 – *Lactiplantibacillus plantarum*). After biopurification of the substrate the proteins were subjected to *in vitro* digestion according to a previously published harmonized static in vitro digestion protocol, INFOGEST[23]. This procedure recapitulates physiological aspects of in vivo digestive processes by subjecting proteins to a sequence of oral, gastric and intestinal conditions including electrolyte, pH and enzymatic processing. We investigated if biopurification affected the susceptibility of proteins to be processed and found that amino acid release, peptide generation and breakdown of intact proteins was largely not affected by a one-hour biopurification process. Only treatment of pea protein with LAB 61 showed a minor, yet not complete, reduction of digestibility.

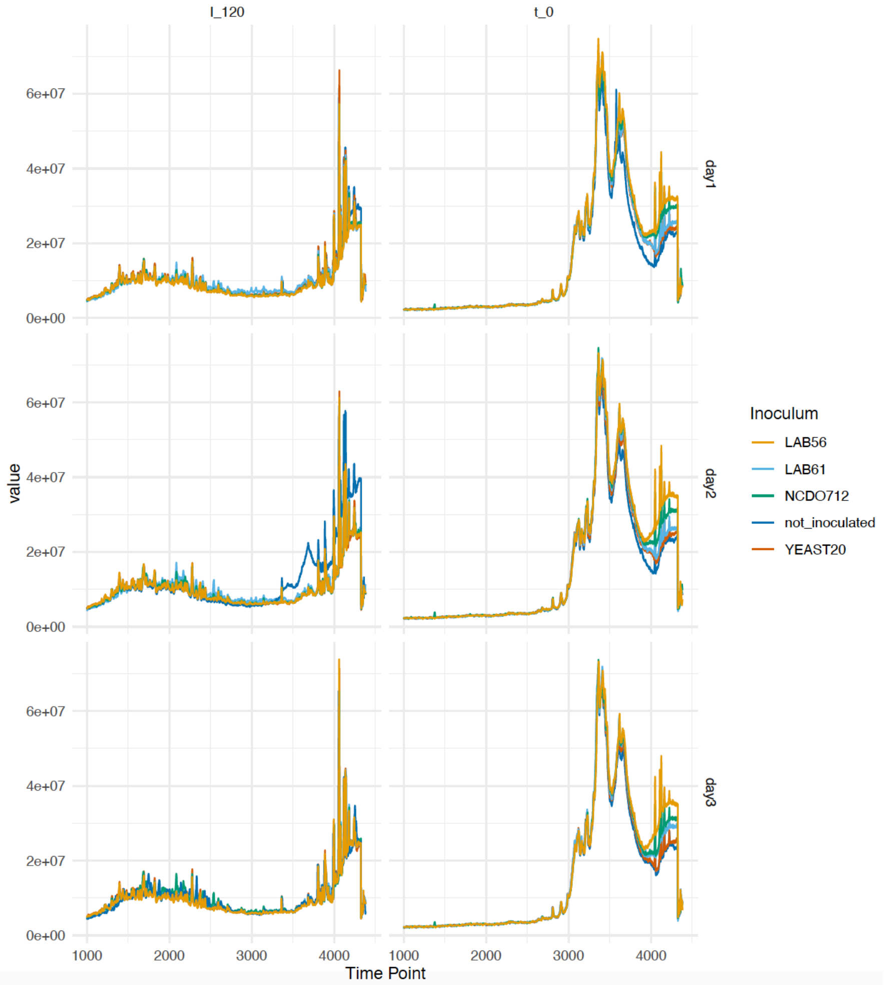

Supplementary Figure 15: Reversed Phase – High Performance Liquid Chromatography-UV-qTOF-MS analysis of pea protein digests. Pea proteins were biopurified for one hour with the indicated strain or incubated at the same conditions without inoculation. Right panels: before digestion, left panels, after digestion. The rows from top to bottom (Day1 – Day3) are independent replicates of the experiment. Note significant formation of peptide fragments at an acquisition time until approximately measurement point 2500.

Amino acid analysis

The determination of amino acids after biopurification (t=0) shows limited differences between the non-biopurified control and the biopurified samples. The main difference is that strain NCDO712 produced ornithine and citrulline. This is a property *L. cremoris* NCDO712 is known for, due to the presence of an arginine deiminase gene cluster[16,24].

During in vitro digestion amino acid released in samples treated with yeast hardly differed from the controls that were not biopurified. Samples treated with either *L. cremoris* NCDO712 or with *Leuconostoc mesenteroides* (LAB61) showed lower concentrations of Met, Thr, His, Ala and Ser. This could be either due to consumption by the organisms or the inhibition of its release during in vitro digestions. For NCDO712 specifically we see again high concentrations of ornithine and citrulline.

Overall, this shows that the type of strain used for biopurification can have an impact on the free amino acid concentrations after in vitro digestion. It also points out that these changes are strain dependent which allows for the control of it during biopurification, through the selection of non- or highly proteolytic strains

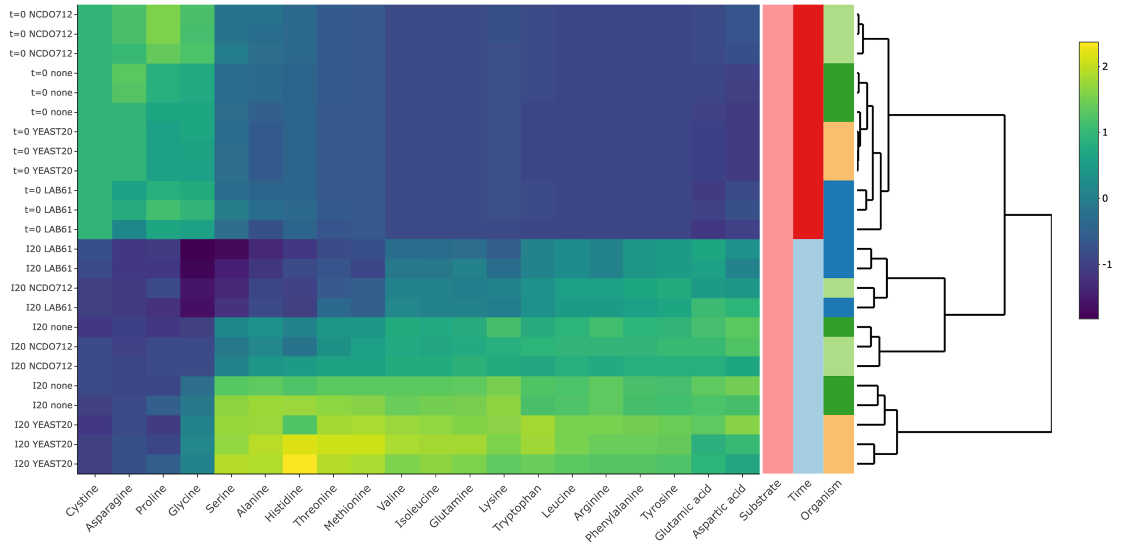

Supplementary Figure 16: Heatmap of the amino acid concentrations measured after biopurification (t=0) and after in vitro digestions (I120) (see description on the left) of pea protein. Biological triplicates are shown individually.

Supplementary information 11: Techno-functionality of biopurified protein ingredients

To confirm for limited changes in techno-functionality, foaming, emulsification, and protein solubility were compared on non-sterilized protein solutions before and after biopurification (Figure 4). Overall, foam overrun, total soluble nitrogen, and emulsion droplet size distribution did not show significant changes. Exception includes pea protein isolate where biopurification by LAB or yeast resulted in 25% increase in foaming ability and stability. Aside from biopurification effect or a lack thereof, the measured techno-functionalities showed significant differences between the various sources.

Foam overrun is high in pea and potato proteins at roughly 100 and 150%, respectively. Low foam overrun at approximately 25% was found in almond and oat. High protein solubility was observed in almond and potato proteins at approximately 50 and 90% solubility, respectively. In contrast, pea (19% solubility) and oat (5% solubility) proteins were poorly soluble. High emulsifying ability was seen in pea and potato proteins, which shows an average droplet diameter of 0.5 μm. Emulsification with oat protein and almond protein results in average droplet diameter of 7.8 μm and 12.2 μm. Following 2-day storage, pea and potato emulsions were stable, while oat and almond emulsions

exhibited phase separation (creaming). Altogether, foaming properties may be slightly improved and other properties remain unaltered by our approach of off-flavour biopurification.

Supplementary Information S12: Supplementary Materials and Methods

Strains and cultivation conditions

Table S7: Species of LAB and yeasts used throughout this study and their corresponding pre-culuture cultivation medium and temperature. Pre-cultures incubations were 24 hours for lactococci, streptococci and lactobacilli and 48 hour for leuconostoc and yeast. Yeast cultures were shaken at 250 rpm. All other cultures were not shaken during incubations.

ID	Species	Cultivation Medium	Cultivation temperature (°C)
**LAB 31**	*Lactococcus lactis*	GM17	30
**LAB 32**	*Lactococcus lactis subps lactis*	GM17	30
**LAB 33**	*Lactococcus lactis*	GM17	30
**LAB 34**	*Leuconostoc pseudomesenteroides*	MRS	30
**LAB 35**	*Streptococcus thermophilus*	GM17	37
**LAB 36**	*Streptococcus thermophilus*	GM17	37
**LAB 37**	*Streptococcus thermophilus*	GM17	37
**LAB 38**	*Streptococcus thermophilus*	GM17	37
**LAB 39**	*Streptococcus thermophilus*	GM17	37
**LAB 40**	*Leuconostoc mesenteroides*	MRS	30
**LAB 41**	*Leuconostoc mesenteroides*	MRS	30
**LAB 42**	*Leuconostoc mesenteroides*	MRS	30
**LAB 43**	*Leuconostoc lactis*	MRS	30
**LAB 44**	*Lactobacillus rhamnosus*	MRS	37
**LAB 45**	*Lactobacillus curvatus*	MRS	37
**LAB 46**	*Leuconostoc paramesenteroides*	MRS	30
**LAB 47**	*Lactobacillus plantarum*	MRS	37
**LAB 48**	*Lactococcus lactis*	GM17	30
**LAB 49**	*Leuconostoc lactis*	MRS	30
**LAB 50**	*Lactococcus lactis subps lactis*	GM17	30
**LAB 51**	*Lactococcus lactis*	GM17	30
**LAB 52**	*Lactococcus lactis subps lactis*	GM17	30
**LAB 53**	*Lactococcus lactis subps lactis*	GM17	30
**LAB 54**	*Lactococcus lactis subps lactis*	GM17	30
**LAB 55**	*Leuconostoc mesenteroides mesenteroides*	MRS	30
**LAB 56**	*Lactobacillus plantarum*	MRS	37
**LAB 57**	*Pediococcus acidilactici*	MRS	30
**LAB 58**	*Lactobacillus helveticus*	MRS	37
**LAB 59**	*Leuconostoc suionicum*	MRS	30
**LAB 60**	*Leuconostoc suionicum*	MRS	30
**LAB 61**	*Leuconostoc mesenteroides mesenteroides*	MRS	30
**LAB 62**	*Lactobacillus paracasei*	MRS	37
**LAB 63**	*Lactobacillus paracasei*	MRS	37
**LAB 64**	*Lactobacillus plantarum*	MRS	37
**LAB 65**	*Lactobacillus curvatus*	MRS	37
**LAB 66**	*Streptococcus thermophilus*	GM17	37

**LAB 67**	*Lactococcus lactis subps cremoris*	GM17	30
**LAB 68**	*Lactobacillus fermentum*	MRS	37
**LAB 69**	*Leuconostoc mesenteroides*	MRS	30
**LAB 70**	*Lactobacillus brevis*	MRS	37
**LAB 71**	*Lactobacillus curvatus*	MRS	37
**LAB 72**	*Lactobacillus curvatus*	MRS	37
**LAB 73**	*Lactobacillus curvatus*	MRS	37
**LAB 74**	*Lactobacillus helveticus*	MRS	37
**LAB 75**	*Lactobacillus helveticus*	MRS	37
**LAB 76**	*Lactobacillus oris*	MRS	37
**LAB 77**	*Lactobacillus parabuchneri*	MRS	76
**LAB 78**	*Lactobacillus paracasei*	MRS	37
**LAB 79**	*Lactobacillus rhamnosus*	MRS	37
**LAB 80**	*Lactobacillus rhamnosus*	MRS	37
**LAB 81**	*Lactobacillus rhamnosus*	MRS	37
**LAB 82**	*Lactobacillus rhamnosus*	MRS	37
**LAB 83**	*Leuconostoc carnosum*	MRS	30
**LAB 84**	*Leuconostoc carnosum*	MRS	30
**LAB 85**	*Leuconostoc carnosum*	MRS	30
**LAB 86**	*Leuconostoc citreum*	MRS	30
**LAB 87**	*Leuconostoc citreum*	MRS	30
**LAB 88**	*Leuconostoc fallax*	MRS	30
**LAB 89**	*Leuconostoc fructosum*	MRS	30
**LAB 90**	*Leuconostoc gasicomitatum*	MRS	30
**LAB 91**	*Leuconostoc gasicomitatum*	MRS	30
**LAB 92**	*Leuconostoc gasicomitatum*	MRS	30
**LAB 93**	*Leuconostoc lactis*	MRS	30
**LAB 94**	*Leuconostoc mesenteroides cremoris*	MRS	30
**LAB 95**	*Leuconostoc mesenteroides dextranicum*	MRS	30
**LAB 96**	*Leuconostoc mesenteroides dextranicum*	MRS	30
**LAB 97**	*Leuconostoc pseudomesenteroides*	MRS	30
**LAB 98**	*Lactobacillus buchneri*	MRS	37
**LAB 99**	*Lactococcus lactis subps cremoris*	GM17	30
**LAB 100**	*Lactococcus lactis subps cremoris*	GM17	30
	Lactococcus cremoris MG1363[16]	CDMpc[25]	30
	Lactococcus cremoris NCDO712[24]	GM17	30
**Yeast 16**	*Zygosaccharomyces rouxii*	GYP	25
**Yeast 17**	*Pichia kluyveri*	GYP	25
**Yeast 18**	*Candida versatilis*	GYP	25
**Yeast 19**	*Dekkera bruxellensis*	GYP	25
**Yeast 20**	*Pichia manshurica*	GYP	25
**Yeast 21**	*Candida zemplinina*	GYP	25
**Yeast 22**	*Torulaspora microellipsoides*	GYP	25
**Yeast 23**	*Dekkera bruxellensis*	GYP	25
**Yeast 24**	*Candida sake*	GYP	25
**Yeast 25**	*Candida etchellsii*	GYP	25

**Yeast 26**	*Brettanomyces custersianus*	GYP	25
**Yeast 27**	*Wickerhamomyces subpelliculosus*	GYP	25
**Yeast 28**	*Zygosaccharomyces bailii var. bailii*	GYP	25
**Yeast 29**	*Torulaspora microellipsoides*	GYP	25
**Yeast 30**	*Pichia spartinae*	GYP	25
**Yeast 31**	*Pichia scutulata*	GYP	25
**Yeast 32**	*Zygotorulaspora florentina*	GYP	25
**Yeast 33**	*Pichia deserticola*	GYP	25
**Yeast 34**	*Wickerhamomyces subpelliculosus*	GYP	25
**Yeast 35**	*Brettanomyces custersianus*	GYP	25
**Yeast 36**	*Zygosaccharomyces kombuchaensis*	GYP	25
**Yeast 37**	*Barnettozyma californica*	GYP	25
**Yeast 38**	*Zygosaccharomyces rouxii*	GYP	25
**Yeast 39**	*Saccharomyces bayanus*	GYP	25
**Yeast 40**	*Saccharomyces pastorianus*	GYP	25
**Yeast 41**	*Saccharomyces kudriavzevii*	GYP	25
**Yeast 42**	*Saccharomyces kudriavzevii*	GYP	25

Semi dynamic in vitro digestion

INFOGEST static in vitro digestion was performed as described before[23]. Prior to digestion, pea protein solutions were subjected to biopurification by LAB61, NCDO712, YEAST 20, and LAB 56, as described. Pea protein solutions at 10% (w/w) were incubated in electrolyte simulated salivary fluid[26] in the presence of salivary amylase (Sigma, cat. no. A1031) for 2 min at 37C in a shaking water bath. Following this step, the gastric phase of digestion was mimicked by incubation for 30 min in electrolyte simulated gastric fluid and pepsin (Sigma, cat. no. P7000) while gradually lowering the pH to 2.0 by titration with 10 M HCl. Finally, small intestinal digestion was addressed by adding electrolyte simulated intestinal fluid containing bile extract (Sigma, cat. no. B3883), and pancreatin (Sigma, cat. no. P7545) for 120 min while increasing the pH to 7.0. Experiments were performed as independent triplicates and enzyme-containing samples in the absence of protein and non-biopurified pea protein solutions were taken as controls.

Mono and di-saccharide analysis

Neutral mono and di-saccharides were determined by high performance ligand exchange chromatography (HPLEC) with refractive index (RI) detection. Proteins and fat in the sample were precipitated by Grimbely Biggs reagents and removed after centrifugation. 25 µL supernatant was injected on a mixed bed guard in series with two Aminex HPX-87P, 300 x 7.8 mm analytical columns (Bio-Rad). The neutral mono and di-saccharides were eluted isocratic with Milli-Q water with a flow rate of 0.400 mL/min. The column oven was held at a temperature of 80°C. Data analysis was done with Chromeleon software version 7.2.9 (ThermoFisher Scientific). Quantitative analyses were performed by using standards of the neutral mono and di-saccharides (Sigma-Aldrich).

Peptide analysis

Prior to analysis by RP-HPLC-UV-QTOF-MS, protein powder/solution was dissolved in a reducing buffer, containing Bis-Tris, 6.0 M urea, trisodium citrate and dithiothreitol (DTT), pH 8.0. The sample was stirred at room temperature (20°C) for 60 min. Prior to application to the RP-HPLC-UV-QTOF-MS column, the sample was filtered through a 0.22 µm filter.
Reversed phase high performance liquid chromatography (RP-HPLC) with high resolution (HR) accurate mass (AM) mass spectroscopic (MS) detection was used for semi-quantitative analyses of peptides and proteins in plant-based protein ingredients. The analyses were performed with a 1260 series degasser (Agilent), 1260 series binary pump (Agilent), 1260 series automatic liquid sampler (Agilent), 1260 series thermostated column compartment (Agilent), 1290 Infinity II UV diode array detector (Agilent) and a G6530B High Resolution (HR) Accurate mass (AM) QTOF mass spectrometer (Agilent). 20 µL sample was injected on a Aeris Widepore 200 Å, 3.6 µm XB-C18, 250 x 2.1 mm analytical column (Phenomenex) kept at 40°C. Peptides and proteins were eluted at a flow rate of 0.200 mL/min with a linear gradient of 0.1% (v/v) trifluoro acetic acid (TFA) in 100% water to 0.08% (v/v) trifluoro acetic acid (TFA) in 10% water + 90% acetonitrile in 80 minutes.
The mass spectrophotometer comprises a quadrupole time of flight (QTOF) equipped with a dual electro spray ionisation (ESI) source. Analyses were carried out in the positive detection mode. The capillary voltage was set to 3500 V. The drying gas was set to 10 L/min and the nebulizer pressure was set to 25 psi to vaporize the effluent. A capillary gas temperature of 325 °C and a fragmentor voltage of 175 V was used.
MS experiments were carried out by scanning the parent ions $(M + xH)^{x+}$ in the range of 100 – 3200 m/z.
Data analysis was done with MassHunter Qualitative Analysis and BioConfirm software version B.07.00 (Agilent).

GC-O-MS

Volatile compounds were extracted from the biopurified pea protein (a 5% w/w protein powder suspension in RO water) by dynamic headspace sampling (Purge & Trap, 150 ml/min He purged through 16 ml of 5% w/w pea protein suspension at 42 °C for 30 min with adsorption tube filled with Carobsieve/Carbotrap®) and also with stir bar sorptive extraction (SBSE, 20 ml of 5% protein powder suspension in water for 4 hours at R.T. under stirring)) and analyzed with gas chromatography coupled to a mass spectrometer (Thermo Finnigan TraceGC ultra, Finnigan Trace MS GC/MS) with an olfactometry port (GC/MS-O).

The collected volatile compounds from both extraction techniques were simultaneously thermally desorbed (15 min at 275 °C with a direct flow of 60 ml/min and refocused at -10 ^{0}C on active PDMS phase) using a Unity (Markes International, Bridgend, UK). After rapidly heating the trap to 300 °C, the compounds were separated on an analytical GC column VF-1ms 60 m×0.25 µm column (Agilent, The Netherlands) with a film thickness of 1 µm. After separation on the GC column, the effluent was split to the MS and to the olfactometry port. Mass spectra were recorded over a range of m/z 25-

250. Identification of volatile compounds was done with commercially available libraries like NIST and Wiley. The olfactometry port was analyzed by 3 experienced test subjects, in duplicate. Audio of the aroma descriptions were recorded (Magix, music cleaning lab 2006 deluxe). A compound was considered identified if the description of the compound, identified by means of structure (MS) and Kovats index, corresponded with the sensory description described in literature. The peak areas obtained from the MS were used for semi-quantitative comparison.

Sensory analysis

Samples for sensory analysis were prepared as an aqueous drink and a milk analogue (an oil in water emulsion). The suspension was prepared by dissolving 3.5% w/v pea protein in water. For the emulsion, 3.5% protein and 1.5% sunflower oil were mixed. Both samples were homogenized with an Utra Turrax homogenizer. The bacterial culture for inoculation was prepared as described above with the difference that the cultivation and washing of the cells was done under food grade conditions. Suspension and emulsion samples were inoculated with an overnight culture at an optical density of 0.1 and the samples were incubated at 30 °C for 24 hours. Control samples were incubated without inoculation.

For sensory evaluation using 'Check All That Applies' (CATA), two sensory experts generated attributes for aroma and flavour in advance. The attributes were offered to 10 panel members who were not trained, but familiar with the evaluation of ingredients or products. As the intensity is not well represented by the CATA analysis, the method was slightly adapted to obtain an indication about the quantitative aspects in addition to the qualitative aspects. Therefore, the attributes were also divided into 4 categories, namely slight, medium, strong, and very strong intensity which corresponded to the scores 1-4 respectively. The samples were offered blind and in random order in a single session. The assessor ticked the box that was considered the most appropriate. The scores were analyzed using a one-way analysis of variance (ANOVA) assuming a normal distribution of the data.

Techno-functionality analysis

Immediately after biopurification as described in the main text, samples were freeze-dried for further analysis.

Protein solubility

The protein powders were dispersed in duplicate in water at 20°C for 120 min at protein concentrations of 20 mg/mL. The dispersions were centrifuged at 20.000 x g for 10 min at 20 °C and the supernatants were collected. The protein content of the supernatants was analyzed by Kjeldahl. The protein solubility (%) was calculated as: 100 * protein concentration in supernatant /protein concentration in protein dispersion.

Emulsification

The protein powders were dispersed in duplicate in water at 20°C for 30 min at protein concentrations of 20 mg/mL. Non-purified sunflower oil (purchased at a local supermarket) was added to each dispersion to a final oil content of 10% and the dispersions were pre-emulsified with an Ultra-Turrax at 12000 rpm for 2 min. The pre-emulsions were subsequently homogenized (table top Panda, GEA) at a pressure of 300/30 bar. Droplet size distributions of the emulsions were determined in duplicate by static light scattering (Mastersizer 3000, Malvern Instruments Ltd., Malvern, UK) at room temperature and using a refractive index of 1.48.

Foaming

The protein powders were dispersed in duplicate in water at 20°C for 30 min at protein concentrations of 40 mg/mL. The dispersions were whipped with an Aerolatte mixer for 70 s and the foamed suspensions were transferred to a graduated cylinder. The height of the foam and liquid layers was determined over time (10 s, 5 min, 15 min and 60 min). The foam overrun (%) was calculated as: (100 * height foam layer at t=x / height foam layer at t=0) – 100.

References

1. Duckham, S. C., Dodson, A. T., Bakker, J. & Ames, J. M. *A comparison of eleven potato cultivars*. doi:10.1002/1521-3803.

2. Mandin, O., Duckham, S. C. & Ames, J. M. Volatile compounds from potato-like model systems. *J Agric Food Chem* **47**, 2355–2359 (1999).

3. Opgrande, J. L. *et al.* Benzaldehyde. *Kirk-Othmer Encyclopedia of Chemical Technology* (2000) doi:10.1002/0471238961.0205142615160718.A01.

4. Nie, S. P. *et al.* Effect of pH, temperature and heating time on the formation of furan in sugar–glycine model systems. *Food Science and Human Wellness* **2**, 87–92 (2013).

5. Duckham, S. C., Dodson, A. T., Bakker, J. & Ames, J. M. *A comparison of eleven potato cultivars*. doi:10.1002/1521-3803.

6. Mandin, O., Duckham, S. C. & Ames, J. M. Volatile compounds from potato-like model systems. *J Agric Food Chem* **47**, 2355–2359 (1999).

7. Books, G. S. Industrial Proteins from Potato Juice. A Review.

8. Erten, E. S. & Cadwallader, K. R. Identification of predominant aroma components of raw, dry roasted and oil roasted almonds. *Food Chem* **217**, 244–253 (2017).

9. Nie, S. P. *et al.* Effect of pH, temperature and heating time on the formation of furan in sugar–glycine model systems. *Food Science and Human Wellness* **2**, 87–92 (2013).

10. Fischer, E., Cachon, R. & Cayot, N. Impact of Ageing on Pea Protein Volatile Compounds and Correlation with Odor. *Molecules* **27**, (2022).

11. Tate & Lyle. Petition to add to the national list 205.606: Oat protein concentrate. (2016).

12. Blue Diamond Growers. GRAS Notice (GRN) No. 918 for Partially Defatted Almond Protein Flour. (2020).

13. Tan, Y., Wannasin, D. & McClements, D. J. Utilization of potato protein fractions to form oil-in-water nanoemulsions: Impact of pH, salt, and heat on their stability. *Food Hydrocoll* **137**, 108356 (2023).

14. Chen, Y. *et al.* Proteome constraints reveal targets for improving microbial fitness in nutrient-rich environments. *Mol Syst Biol* **17**, 1–13 (2021).

15. Goel, A. *et al.* Protein costs do not explain evolution of metabolic strategies and regulation of ribosomal content: Does protein investment explain an anaerobic bacterial Crabtree effect? *Mol Microbiol* **97**, 77–92 (2015).

16. Wegmann, U. *et al.* Complete genome sequence of the prototype lactic acid bacterium Lactococcus lactis subsp. cremoris MG1363. *J Bacteriol* **189**, 3256–3270 (2007).

17. Endo, A. *et al.* Fructophilic lactic acid bacteria, a unique group of fructose-fermenting microbes. *Appl Environ Microbiol* **84**, 1290–1308 (2018).

18. Bjorkroth, K. J. *et al.* Characterization of Leuconostoc gasicomitatum sp. nov., Associated with Spoiled Raw Tomato-Marinated Broiler Meat Strips Packaged under Modified-Atmosphere Conditions. *Appl Environ Microbiol* **66**, 3764 (2000).

19. Hinterholzer, A., Lemos, T. & Schieberle, P. Identification of the key odorants in raw French beans and changes during cooking. *European Food Research and Technology* **207**, 219–222 (1998).

20. Kaneko, S., Kumazawa, K. & Nishimura, O. Studies on the key aroma compounds in soy milk made from three different soybean cultivars. *J Agric Food Chem* **59**, 12204–12209 (2011).

21. Xu, M., Jin, Z., Gu, Z., Rao, J. & Chen, B. Changes in odor characteristics of pulse protein isolates from germinated chickpea, lentil, and yellow pea: Role of lipoxygenase and free radicals. *Food Chem* **314**, (2020).

22. Fischer, E., Cayot, N. & Cachon, R. Potential of Microorganisms to Decrease the 'Beany' Off-Flavor: A Review. *J Agric Food Chem* **70**, 4493–4508 (2022).

23. Brodkorb, A. *et al.* INFOGEST static in vitro simulation of gastrointestinal food digestion. *Nat Protoc* **14**, 991–1014 (2019).

24. Tarazanova, M. *et al.* Plasmid complement of Lactococcus lactis NCDO712 reveals a novel pilus gene cluster. *PLoS One* **11**, e0167970 (2016).

25. Price, C. E. *et al.* Adaption to glucose limitation is modulated by the pleotropic regulator CcpA, independent of selection pressure strength. *BMC Evol Biol* **19**, 15 (2019).

26. Mulet-Cabero, A.-I. *et al.* A standardised semi-dynamic in vitro digestion method suitable for food – an international consensus. *Food Funct* **11**, 1702–1720 (2020).
